# In silico analysis and expression profiling of S-domain receptor-like kinases (SD-RLKs) under different abiotic stresses in *Arabidopsis thaliana*

**DOI:** 10.1186/s12864-021-08133-9

**Published:** 2021-11-12

**Authors:** Raju Mondal, Subhankar Biswas, Akanksha Srivastava, Suvajit Basu, Maitri Trivedi, Sunil Kumar Singh, Yogesh Mishra

**Affiliations:** 1 Department of Botany, Centre of Advanced Study in Botany, Institute of Science, Banaras Hindu University, Varanasi, Uttar Pradesh 221005 India; 2 Current address: Mulberry Tissue Culture Lab, Central Sericultural Germplasm Resources Center, Central Silk Board-Ministry of Textiles (GoI), Hosur, Tamil Nadu 635109 India; 3 Plant Cell and Molecular Biology Lab, Department of Botany, Faculty of Science, The MS University of Baroda, Vadodara, Gujarat 390 002 India

**Keywords:** Abiotic stresses, Computational analysis, Gene expression, S-domain receptor-like kinases, Signaling

## Abstract

**Background:**

S-domain receptor-like kinases (SD-RLKs) are an important and multi-gene subfamily of plant receptor-like/pelle kinases (RLKs), which are known to play a significant role in the development and immune responses of *Arabidopsis thaliana*. The conserved cysteine residues in the extracellular domain of SD-RLKs make them interesting candidates for sensing reactive oxygen species (ROS), assisting oxidative stress mitigation and associated signaling pathways during abiotic stresses. However, how closely SD-RLKs are interrelated to abiotic stress mitigation and signaling remains unknown in *A. thaliana*.

**Results:**

This study was initiated by examining the chromosomal localization, phylogeny, sequence and differential expression analyses of 37 SD-RLK genes using publicly accessible microarray datasets under cold, osmotic stress, genotoxic stress, drought, salt, UV-B, heat and wounding. Out of 37 SD-RLKs, 12 genes displayed differential expression patterns in both the root and the shoot tissues. Promoter structure analysis suggested that these 12 SD-RLK genes harbour several potential *cis*-regulatory elements (CREs), which are involved in regulating multiple abiotic stress responses. Based on these observations, we investigated the expression patterns of 12 selected SD-RLKs under ozone, wounding, oxidative (methyl viologen), UV-B, cold, and light stress at different time points using semi-qRT-PCR. Of these 12 SD-SRKs, the genes At1g61360, At1g61460, At1g61380, and At4g27300 emerged as potential candidates that maintain their expression in most of the stress treatments till exposure for 12 h. Expression patterns of these four genes were further verified under similar stress treatments using qRT-PCR. The expression analysis indicated that the gene At1g61360, At1g61380, and At1g61460 were mostly up-regulated, whereas the expression of At4g27300 either up- or down-regulated in these conditions.

**Conclusions:**

To summarize, the computational analysis and differential transcript accumulation of SD-RLKs under various abiotic stresses suggested their association with abiotic stress tolerance and related signaling in *A. thaliana*. We believe that a further detailed study will decipher the specific role of these representative SD-RLKs in abiotic stress mitigation vis-a-vis signaling pathways in *A. thaliana*.

**Supplementary Information:**

The online version contains supplementary material available at 10.1186/s12864-021-08133-9.

## Background

Receptor like kinases (RLKs), a multi-gene family, represent the largest class of protein kinases and have been reported to play crucial roles in plant growth, development, hormone perception, and stress-responsive signaling [1–3]. In *Arabidopsis thaliana*, RLK represents a large gene family with > 600 members that form ~ 2.5% of its protein-coding genes [4]. Typically, RLKs contain an intracellular kinase domain, a transmembrane domain, an extracellular domain, and a signal peptide. The extracellular ligand-binding domain perceives signals and subsequently transmits to an intracellular substrate protein via phosphorylation [5]. The family members of RLK greatly vary in their extracellular domain organization. Based on the sequence identity of extracellular domain organization, 16 subfamilies of RLKs, which includes C-type lectin, CRINKLY-like (CR-like), *Catharanthus roseus*-like (CrRLK-like), extensin-like, leaf rust-like (LRK), legume lectin, leucine-rich repeats (LRR), lysine motif (LysM), proline-rich extension like (PERK), receptor-like cytoplasmic kinases (RLCKs), receptor-like kinase in flowers (RKF), thaumatin, self-incompatibility domain (S-domain), the domain of unknown function 26 (DUF26), unknown receptor kinase (URK), and wall-associated kinase (WAK) have been classified to date [6].

Despite the considerable number of RLK family members in *A. thaliana*, few have been functionally characterized and reported to be associated with several biological functions such as development, innate immunity, self-incompatibility, and stress responses [2, 7]. The members of RLK family whose roles have been identified mostly belong to the subfamily of LRR [3]. The examples include CLAVATA1, which functions in shoot apical meristem maintenance [8]; BRASSINOSTEROID INSENSITIVE 1 (BRI1), which mediates brassinosteroid signalling [9]; ERECTA, which involves determining organ shape [10, 11]; and PnLRR (from an Antarctic moss *Pohlia nutans*), which confers abiotic stress tolerance [12]. In addition to LRR, few members of other RLKs subfamilies such as CR-like, DUF26, lectin type, LRK, thaumatin, and a cold-responsive protein kinase 1 (CRPK1) were reported to mediate and regulate various biotic and abiotic stress response by participating in different signal transduction pathways [7, 13–16].

Cysteine-rich repeats RLKs (CRKs), known as DUF26, are most responsive against a wide range of biotic and abiotic stresses [17–19]. The extracellular domains of most CRKs contain two copies of a DUF26 motif with three conserved cysteine residues in a C-8X-C-2X-C configuration [2]. The conserved cysteine residues in each DUF26 domain may form disulphide bridges and are hypothesized as potential targets for thiol redox regulation [2, 11, 14, 20]. In addition to DUF26, cysteine-rich ectodomain is reported in few other subfamilies of RLKs such as S-domain, thaumatin, and LRK [2]. Simultaneously, in response to ROS, few of them have been reported to be transcriptionally induced [16].

S-domain RLKs (SD-RLKs) comprise one of the largest subfamilies with 39 members in *A. thaliana* (with two exceptions) and 147 members in rice [2, 4, 21]. The function of its closest relative, i.e., S-locus receptor like kinase (SRKs) has been documented in the female determinant of specificity in the self-incompatibility responses of crucifers [22, 23]. However, in *Brassica oleracea*, SRK transcripts were reported to be induced by wounding and bacterial infections [24]. This suggests that their roles are possibly not limited to self-incompatibility. In *A. thaliana*, functions of few SD-RLKs such as ARK2 and ARK3 have been characterized, which indicates their function in plant growth or development [25]. Furthermore, S-domain receptor kinase1–6 (SD1–6 or ARK2) is reported to regulate auxin-mediated lateral root development under a phosphate-starved condition [26] and SD1–29 (AT1g61380), known as LIPOOLIGOSACCHARIDE-SPECIFIC REDUCED ELICITATION (LORE), senses low complex metabolites of bacteria to induce immune response [27]. The function of a few SD-RLKs in rice such as OsSIK2, have been characterized, which was reported to be involved in abiotic stress tolerance and delaying the dark-induced leaf senescence [28]. Moreover, OsESG1 was reported to be involved in the early crown root development and drought resistance [29].

Under selection pressure, *A. thaliana* has transformed into a self-compatible species because of the loss of function of S-locus genes during the course of evolution [30]. *A. thaliana* is a self-fertile plant and hence the functionality of SD-RLKs is not only restricted to its reproductive tissues. Therefore, SD-RLK genes might be involved in other functions beyond the self-incompatibility responses. Furthermore, the presence of cysteine residues in their extracellular domain suggests the role of SD-RLKs as redox sensors vis-a-vis abiotic stress mitigation and related signaling. However in *A. thaliana*, how closely SD-RLKs are interrelated to abiotic stress mitigation and associated signaling is not clear.Therefore, in the present study, we investigated the role of SD-RLKs in *A. thaliana* in response to various abiotic stresses. The gene sequences of 37 SD-RKLs from *A. thaliana* were first retrieved and their chromosomal localization, phylogeny and sequence analysis were done. Furthermore, the expression patterns of these 37 genes were analyzed using publicly accessible microarray datasets from both the root and the shoot tissue under various abiotic stresses. Based on microarray**-**based gene expression analysis, 12 potential SD-RLK genes were chosen and subsequently online databases/bioinformatics tools were used to analyze conserved motifs and CREs (*cis*-regulatory elements) in their promoter regions. The expression patterns of these 12 SD-RLK genes were investigated by semi-qRT-PCR under ozone, wound, oxidative (methyl viologen, i.e. MV), UV-B, cold, and light stress. To corroborate the differential transcript accumulation of semi-qRT-PCR results, qRT-PCR analysis was also conducted to identify the potential SD-RLKs under these stresses. The present study will lay the foundation for further research on the function of SD-RLK genes in responses to abiotic stresses in *A. thaliana*.

## Methods

### Sequence retrieval and chromosomal localization

According to Shiu and Bleecker et al. [4], a total of 39 genes encoding SD-RLKs (with two exceptions, i.e. one pseudo and one duplicate gene) were identified in *A. thaliana* (Additional file 1: Table S1). Thus, the emphasis of our investigation was on 37 SD-RLK genes. The gene and protein sequences of SD-RLKs were retrieved from the TAIR database (www.arabidopsis.org [31];). The physical location of these genes on *A. thaliana* genome was determined by TAIR chromosome map tools (www.arabidopsis.org/jsp/ChromosomeMap/tool.jsp).

### Sequence alignment and evolutionary relationship

To understand the evolutionary relationship among identified SD-RLKs, protein sequences were subjected to Clustal Omega program (www.ebi.ac.uk/Tools/msa/clustalo/ [32];) pursuing default parameters and phylogenetic tree was constructed using the iTOL browser (https://itol.embl.de/ [33];). The positions of the trans-membrane domain of each SD-RLK were identified using HMMER (http://hmmer.org/ [34];) and the extracellular peptide sequences were used for the alignment. The sequence alignment was performed using Jalview 2.11.1.3 (https://www.jalview.org/) using the multiple sequence alignment program Tcoffee with default settings [35]. All cysteine residues were marked with red colour for explaining conservativeness (Additional files 1: Table S1 and Additional files 2: Fig. S1). DiANNA 1.1 (http://clavius.bc.edu/~clotelab/DiANNA [36];) web server was employed for extracellular disulfide bond prediction. Note that only the extracellular disulphide bonds with a bonding score of > 0.7 were considered for analysis.

### Conserved motif and domain analysis of SD-RLKs encoded proteins

The conserved motif sequences and their location across the protein length of SD-RLKs were identified from MeMe suite database (http://meme-suite.org [37];) and visualized with TBtools software version 0.665 (https://github.com/CJ-Chen/TBtools [38];). In addition, conserved domains were predicted using NCBI *Batch CD-Search* with Pfam live search (https://www.ncbi.nlm.nih.gov/Structure/bwrpsb/bwrpsb.cgi [39];). After retrieving hitdata, it was subjected to visualization using TBtools software version 0.665 (https://github.com/CJ-Chen/TBtools [38];).

### Gene expression profiling of 37 SD-RLKs using publicly accessible microarray datasets

To understand the differential gene expression pattern of SD-RLKs under different abiotic stresses, microarray**-**based gene expression data for 37 SD-RLKs of *A. thaliana* were used. A total of nine abiotic stress conditions, including cold, osmotic, salt, drought, genotoxic, oxidative, UV-B, wounding, and heat stress, were chosen to investigate the differential expression of these SD-RLKs under these stresses. Therefore, nine AtGeneExpress (Stress Series) samples were downloaded from Bio-Analytic Resource, e-Northerns w. Expression Browser (http://bar.utoronto.ca [40];), and the array was normalized with a TGT value of 100 using the Gene Chip Operating Software (GCOS). The data-sets comprised of an average of replicate treatments relative to average of appropriate control and output values in the table and image were log2-transformed ratios. For DEG analysis, data-sets comprised of seven data-point of nine samples were extracted in notepad and then the differential expression of 37 genes was depicted in a heatmap using default criteria of TBtools software version 0.665 (https://github.com/CJ-Chen/TBtools [38];). According to experimental information, *A. thaliana* wild type (col-0) seeds were spread in Magenta boxes containing MS-medium. Following 2 days of 4 °C (dark) incubation, samples were transferred to the growth chamber (16/8 h photoperiod, 24 °C temperature, 50% relative humidity, and light intensity of 150 μEinstein/cm^2^ sec). After 11 days, plants were cultured in liquid MS-media. 16-days-old seedlings (at 3 h of light period) were exposed to above-mentioned stresses for different time points such as 0.5, 1, 3, 6, 12, 24 h, with control samples including 0 h. Roots and shoots were prepared separately for tissue-specific expression, and all treatments were performed on the same batch of seedlings.

### Promoter structure analysis and functional annotation of *cis*-regulatory elements (CREs) in 12 SD-RLKs

To better understand the transcriptional regulation of 12 most responsive SD-RLKs (selected from microarray**-**based gene expression analysis) under abiotic stress conditions, 500 bp-sized nucleotide sequences upstream of their transcription start site (TSS), were used for the prediction of putative CREs using PlantCARE database (http://bioinformatics.psb.ugent.be/webtools/plantcare/html [41];). The identified CREs, their distribution pattern throughout the 12 SD-RLK genes, and their functional annotation were visualized using TBtools software version 0.665 (https://github.com/CJ-Chen/TBtools [38];).

### *A. thaliana* growth conditions and stress treatments

The seeds of *A. thaliana* ecotype Columbia-0 (Col-0) were obtained from the Arabidopsis Biological Resource Centre (ABRC), The Ohio State University, USA [42]. Arabidopsis seeds were sterilized using freshly prepared 5% calcium hypochlorite for 10 min and plated on half-strength MS medium supplemented with 1% sucrose and maintained for 3 days at 4 °C. The cultured plates were subsequently transferred to plant growth chamber (Conviron, Adaptis 1000) having a temperature of 23 °C, bright fluorescent light of 150 μmol quanta m^− 2^ s ^− 1^, relative humidity of 65%, and a 16 h light: 8 h dark cycle.

For stress treatments, 10-day-old seedlings (+ 3 d stratification) were exposed to six abiotic stresses i.e. ozone (40 ppb), wounding, oxidative stress (*MV-* 25 μM), cold (8 °C), UV-B light (0.99 W m^− 2^ s^− 1^), and light (500 μmol quanta m^− 2^ s^− 1^) for 2, 6, and 12 h. The doses of each stresses were selected on the basis of earlier studies performed in *A. thaliana* [43–47]*.* In addition to each stress treatment, a similar set of seedlings were maintained in its normal growing condition and used as control.

### Detection of classic stress markers

The selected abiotic stresses are known to enhance the production of ROS, such as singlet oxygen superoxide radical, hydrogen peroxide, and hydroxyl radical in plants [43–47]. In order to assess the stress status of the plants during exposure to the aforesaid abiotic stresses, presence of two classical stress markers i.e. superoxide radical and hydrogen peroxide were assessed using nitroblue tetrazolium (NBT) and 3, 3’diaminobenzidine (DAB) staining methods, respectively, in the control and stress exposed seedlings at selected time points, namely, 0 (control), 2, 6, and 12 h.

According to the procedure of Ramel et al. (2009) [48], NBT was employed to detect superoxide radical in situ. Seedlings were submerged in 3.5 mg ml^− 1^ NBT dissolved in 10 mM potassium phosphate buffer (pH 7.5) with 10 mM NaN_3_ and then vacuum infiltrated. After infiltration, seedlings containing plates were kept in the dark for 45 min at room temperature, then in the light for 15 min to observe if a blue formazan precipitate appeared. The blue colored seedlings were bleached with acetic acid-glycerol-ethanol (1:1:3) (V:V:V) at 100 °C for 5 min.

Furthermore, DAB was employed to detect hydrogen peroxide in situ, as described by Zhang et al. (2012) [49]. Seedlings were submerged in 50 mM phosphate buffer with 1.25 mg ml^-1^ DAB (pH 7.0) and then vacuum infiltrated. After infiltration, seedlings with plates were maintained in the dark for 8 h before being exposed to white light (80 mol m^− 2^ s^− 1^) to achieve a brown color. The bleaching procedure was identical to the NBT staining procedure. The photos were taken with a stereo zoom microscope assisted with a Magcam DC5 camera (Magnus, MSZ-TR).

### RNA isolation and cDNA synthesis

Total RNA was extracted from the control and stress exposed seedlings at selected time points, namely, 0 (control), 2, 6, and 12 h. Seedlings were ground under liquid nitrogen and the tissue powder was transferred to the RNase free micro-centrifuge tube. As per the manufacturer’s instructions of RNeasy plant mini kit (Qiagen, USA), RNA was isolated from the samples. Subsequently, the quantity and quality of isolated RNA was assessed using a spectrophotometer (Nanodrop 2000, Thermo Fisher Scientific, Waltham, MA, USA) and formaldehyde-based gel electrophoresis, respectively. For cDNA synthesis, 1 μg of total RNA was reverse-transcribed in a total volume of 20 μl using Revert Aid First Strand cDNA Synthesis Kit (Fermentas Life Sciences, USA) using oligo (*dT*) primers as per the manufacturer’s instructions.

### Differential gene expression analysis of 12 selected SD-RLKs under six abiotic stresses

To investigate the differential temporal expression patterns of 12 selected genes of SD-RLKs against six abiotic stresses, we used semi-qRT-PCR and afterward four of them were verified by qRT-PCR using three biological replicates. Semi-qRT-PCR was performed in a reaction mixture of 25 μl containing 12.5 μl PCR Master Mix (2X) (Thermo Scientific, USA), 1 μl of cDNA (50 ng), 10 pmol of each primer, and nuclease-free water using Bio-Rad MyCycler™ system (Bio-Rad, USA). Depending on gene-specific amplification, annealing temperature was varied from 55 to 59 °C. The amplification of *AtUBQ5* and *AtEF1α* was performed as an internal control. For gel electrophoresis, 10 μl of PCR products were loaded in 1.5% agarose gel containing 0.5 μg of ethidium bromide per 100 ml gel volume in tris-acetate-ethylenediaminetetraacetic acid (TAE) buffer (pH 8) and run at 50 V for 30 min. Electrophoretic gels were then scanned in Image Quant LAS500 (GE Healthcare). The product size of each PCR amplicon was determined by comparing the corresponding band of the 50 bp DNA ladder (Genei, Bangalore).

Further verification of expression of four most potential SD-RLK genes was done by qRT-PCR using CFX-96 Real-time PCR Detection System (Bio-Rad, USA). Reactions were performed in a total volume of 20 μl using 50 ng of cDNA, 10 pmol of forward and reverse primers, and 10 μl of 1× Sso Fast Eva Green qPCR Supermix (Bio-Rad, USA). The cycling conditions were as per the manufacturer’s protocol with varying annealing temperature of gene-specific primer sets. The specificity of PCR was determined using the melt curve analysis of amplified products. The threshold cycle (Ct) was automatically determined for each reaction using the system set with default parameters. Gene-specific and two reference primers (*AtUBQ5* and *AtAPT1*) used were designed to produce 100–150 bp PCR products. The gene expression levels were normalized against internal references. The fold difference of each amplified product in the samples was calculated using the 2^−ΔΔCt^ method. The list of all primers used for semi-qRT-PCR and qRT-PCR is given in Additional files 3: Table S2.

### Statistical analysis

Data were expressed as mean ± standard deviation (SD) of at least three biological replicates. To determine significant differences in control and stress treated samples, the results of expression data were statistically analysed using one-way analysis of variance (ANOVA), followed by Tukey’s post-hoc test using SigmaPlot 12 (*p* < 0.05) [50].

## Results

### Chromosomal localization, phylogenetic relationship and sequence analysis of SD-RLKs

**Fig. 1:**
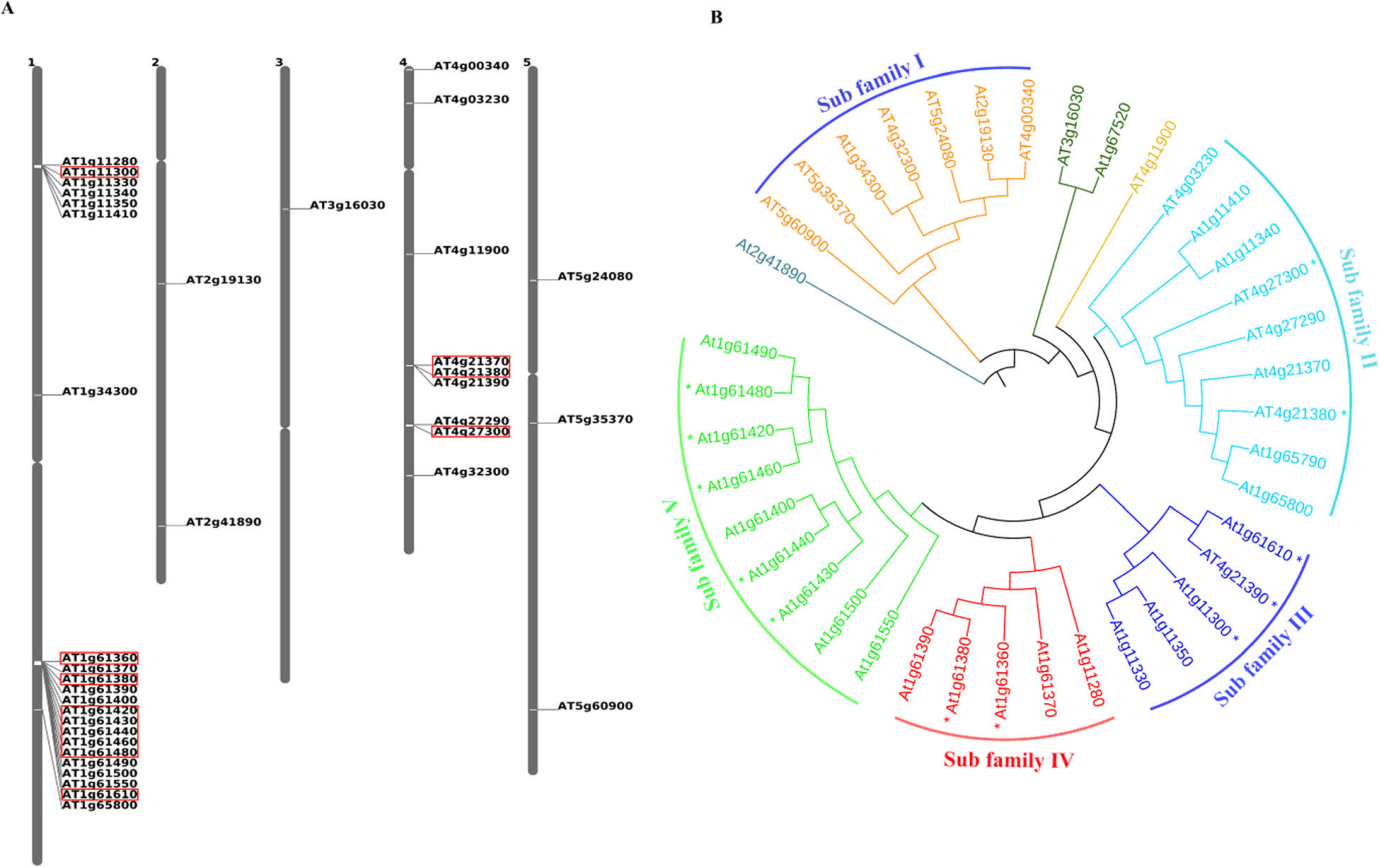
Chromosomal localization and phylogenetic relationship of SD-RLKs. **A.** Distribution of SD-RLK genes on different chromosomes of *A. thaliana*. The chromosomal numbers are shown above each chromosome, and the SD-RLK gene names are shown to the right of each chromosome. The red box encircling SD-RLK genes exhibited a potential differential expression pattern under various abiotic stresses when in silico analysis of microarray data was done. **B.** SD-RLKs peptide sequences of SD-RLKs in *A. thaliana* were aligned by the Clustal Omega and phylogenetic tree of peptide sequences were generated by using iTOL server with neighbour joining method based on 1000 bootstrap values. In the phylogenetic tree, 37 SD-RLK genes of *A. thaliana* were clustered in five major subfamilies (subfamily I, II, III, IV, and V)

Gene localization using chromosome mapping demonstrated that the SD-RLK subfamily is distributed on all the chromosomes; however, most of the members of this family are present in chromosome 1 and 4. Moreover, only one member of this subfamily is confined on chromosome 3, two members on chromosome 2, and three members on chromosome 5 (Fig. 1A). Out of these 39 genes (Additional file 1: Table S1), At4g21370 was identified as a pseudo gene because of the presence of the premature stop codon [51]. Recently, certain deviations were noticed again in the few members of SD-RLKs in *A. thaliana*; e.g., AT1g67520, AT3g16030, At5g60900, At4g32300 and At5g35370 showed that these genes lack the S-domain/SLG domain, although B-lectin domain and PAN domain were present [52, 53].

The chromosomal distribution patterns of genes of the SD-RLKs also demonstrated that the six and 14 genes were separately clustered together on both arms of chromosome 1, suggesting the possible occurrence of multiple tandem duplication events [54]. Similarly, on the long arm of chromosome 4, tandem duplication events might has happened as three and two members of SD-RLK subfamily are located in two small gene clusters, respectively.

Next, a phylogenetic tree of 37 SD-RLKs peptide sequences of *A. thaliana* was constructed to identify the evolutionary relationship between the encoded proteins (Fig. 1B). As per the topological structure of the phylogenetic tree, all SD-RLKs proteins are clustered into five major subfamilies. Among the five sub-families, the larger subfamily II and V are having 9 members of SD-RLKs each. The comparatively medium-size subfamily I comprises seven members and smaller subfamilies III and IV comprise five members each. A single member At2g41890 and At4g11900 could not cluster in any group reported to be phylogenetically distinct.

For sequence analysis, the ectodomain protein sequences of 37 SD-RLKs were used for alignments. At1g67520 was ignored from this study because of the lack of extracellular domains. The study revealed that rather than having highly diverse numbers of cysteine residues in extracellular domains, 12 cysteine residues are highly conserved in the analyzed SD-RLKs (Additional file 1: Table S1). Most ectodomain cysteines had the potential ability to form intra-molecular disulphide bridges. The presence of this strict conservation of cys residues and their bond forming ability suggests a preserved and significant role of them in the function of these proteins (Additional file 2: Fig. S1).

### Conserve motifs and domains organization of SD-RLKs

**Fig. 2:**
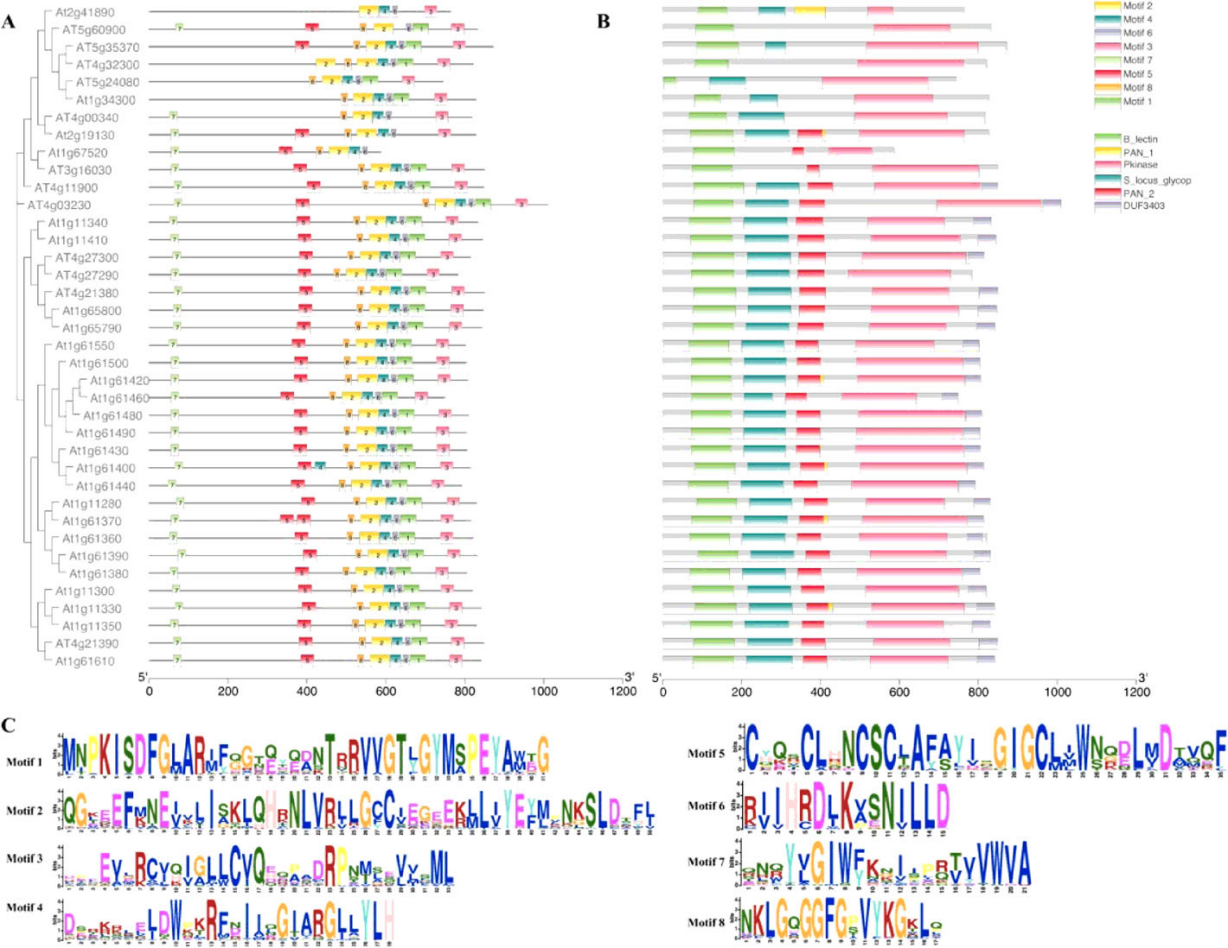
**A.** Pictorial representation of eight specific conserved motif sequences and their location across the protein length in SD-RLKs. **B**. Visualization of six conserved domains namely, B_lectin, PAN_1, PAN_2, Pkinase, S_locus_glycop and DUF3403 in SD-RLKs encoded proteins **C**. Weblogo analysis of amino acid variability and conservation present in the identified conserved motifs of SD-RLKs. The height of the stack is proportional to the conservation of the representative residue

According to the MEME suite study, SD-RLK proteins have varying numbers and sizes of conserved motifs, ranging from 1 to 8 (Fig. 2A). Motifs 1, 2, and 5 are larger in size in comparison to the other motifs. Furthermore, structural predictions showed that C-terminal motifs of *A. thaliana* SD-RLK proteins are more constrained than N-terminal motifs, since At2g41890, At5g35370, At1g34300, At4g32300, and At5g24080 proteins lack N-terminal conserved motifs that are significantly distinct from other members. Their C-terminal portions, on the other hand, shared motifs like motif 3, motif 2, and motif 1. We found that the motifs of SD-RLK proteins belonging to the same subfamily are not similar in terms of sequence composition and relative position (Fig. 2A and C). For example, the motifs in SD-RLK proteins in subfamily-V (9 proteins, At1g61550 to At1g61440) are not in the same place (Fig. 2A).

The domain analysis of 38 SD-RLK proteins revealed that all of them contain B_lectin domain and PAN domain (except six members from At5g60900 to At4g00340) (Fig. 2B). The S locus glycop domain is present in all except At5g60900, At4g32300, At1g67520, and At3g16030. Because the Pkinase domain is present in all members of this subfamily, they are all classified as protein kinases (Fig. 2B).

### Microarray-based gene expression profiling suggested the modulation of SD-RLK genes expression under different abiotic stresses

**Fig. 3:**
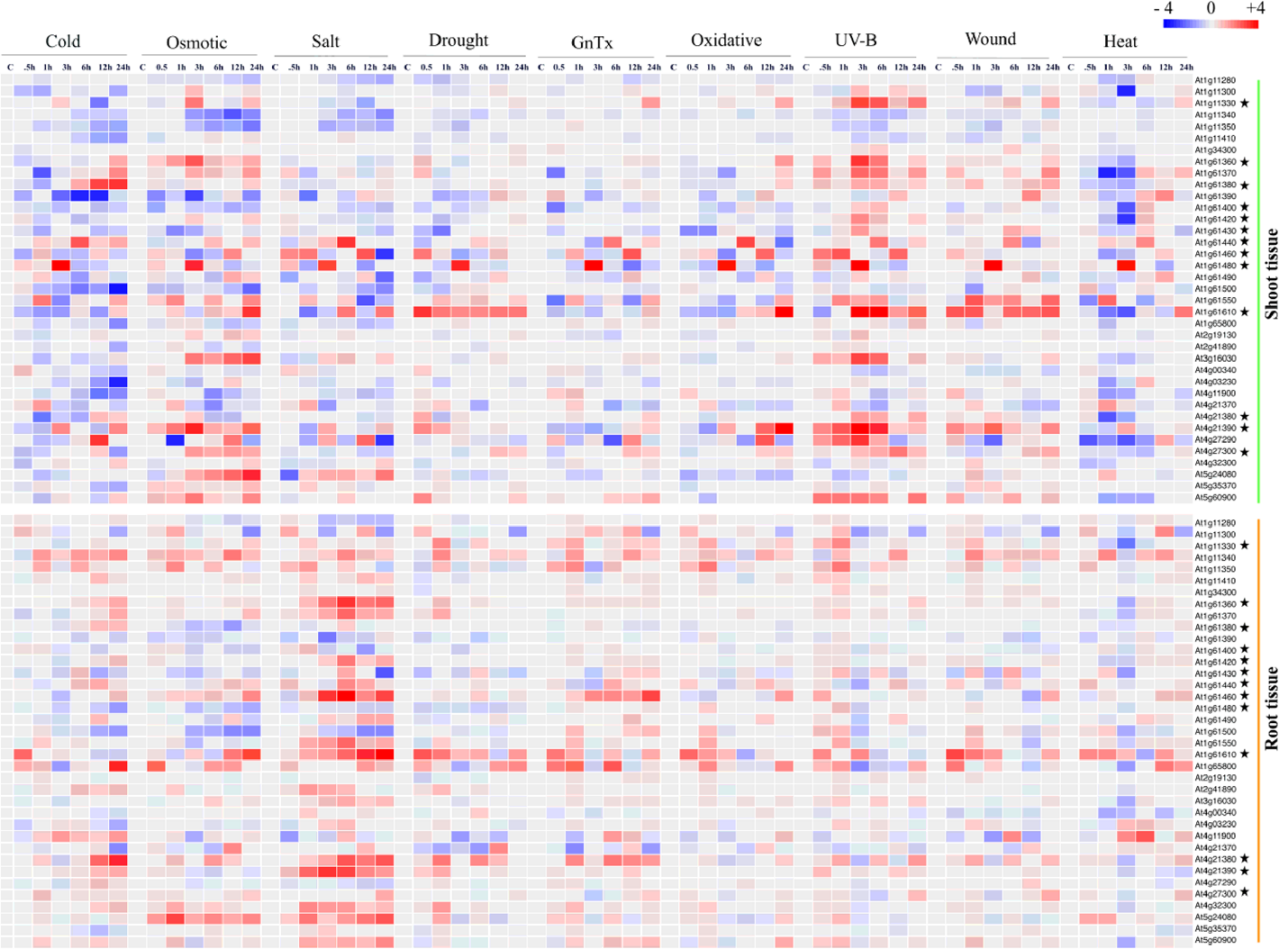
Heatmap visualization of 37 SD-RLK genes expression of *A. thaliana* in response to cold, osmotic, drought, salt, UV-B, heat, wound, and genotoxic stress conditions. The publicly available microarray data for expression of 37 SD-RLK genes from shoot and root tissue under various stress conditions were used to generate heatmap. The colour bar exhibits fold change in gene expression, red colour representing highest level of expression and blue colour signifies lowest level of expression. Genes with promising expression pattern is denoted by star mark (*)

According to the literature assessment, there was a high possibility that the transcriptional regulation of 37 SD-RLK genes in *A. thaliana* is influenced by different abiotic stresses [2, 28]. Thus, we performed expression analysis based on the microarray datasets of all 37 SD-RLK genes across a wide range of abiotic stresses, namely, cold, osmotic, salt, drought, genotoxic, oxidative, UV-B, wound, and heat stress. The heatmap (Fig. 3) depicts the results of this analysis, which show that members of the SD-RLKs were differentially expressed under diverse abiotic stresses. The detailed assessment of the expression profile from the microarray datasets revealed that the majority of the genes had intermediate level expression in both root and shoot tissues; nevertheless, 12 genes exhibited strong expression during different time points of exposure to all abiotic stresses in both root and shoot tissues. Moreover, their expression levels were maintained consistently with the exposed time points (Fig. 3, star marked). The 12 shortlisted genes (AGIs) on the basis of microarray based expression profiling were At4g27300, At1g61440, At4G21390, At1g61610, At1G11330, At1G61380, At1G61460, At1G61430, At4G21380, At1G61360, At1G61480, and At1G61420.

### The promoter region of 12 selected SD-RLKs are enriched with several potential abiotic stress-responsive CREs

**Fig. 4:**
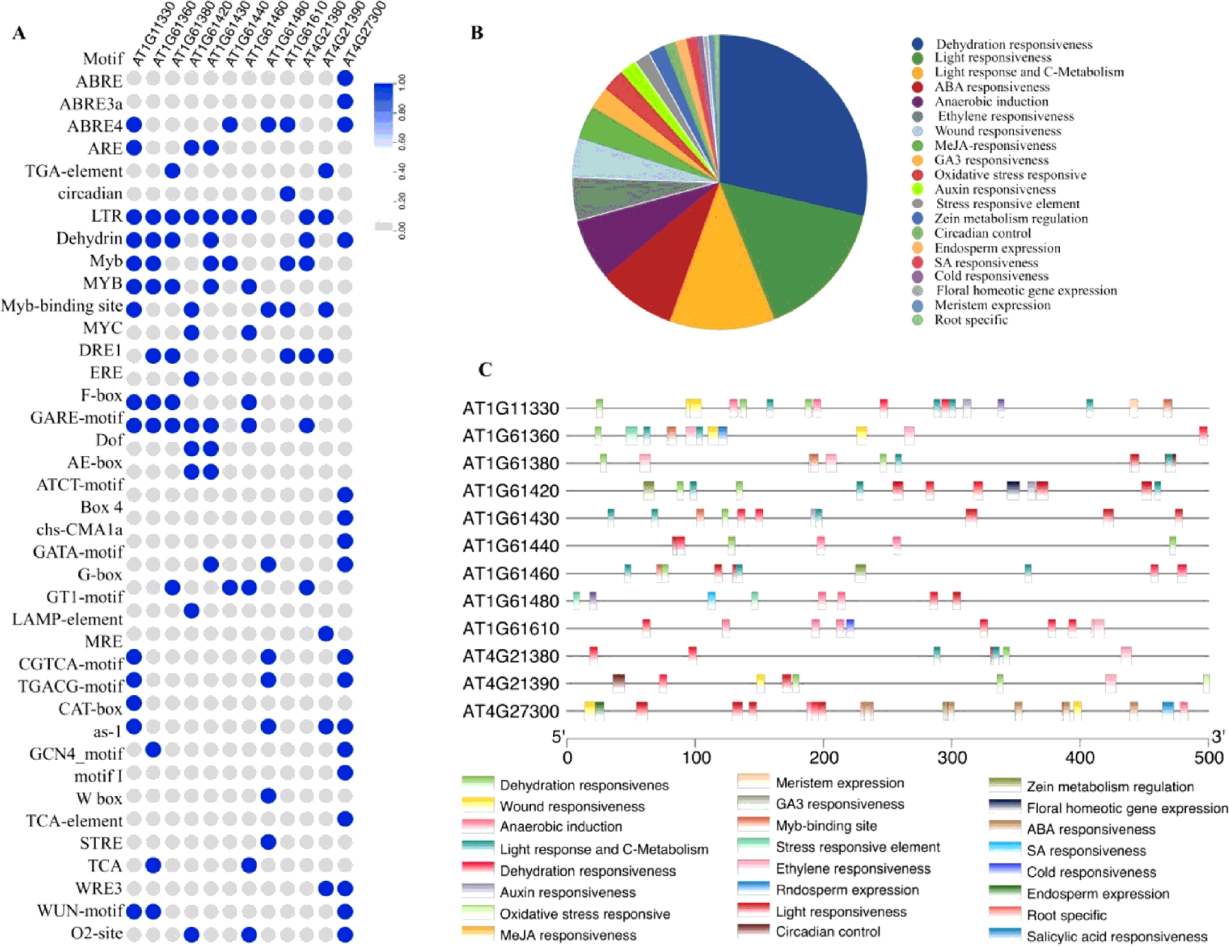
**A.** Dot-plot representation of c*is*-acting regulatory elements (CREs) in promoter regions of 12 selected SD-RLK genes. Blue and gray circle indicates presence and absence of CREs. **B.** Proportional distribution patterns of identified developmental and stress responsive CREs in Pie chart. **C.** Functionally annotated CREs distribution patterns over the promoter length (500 bp) of selected SD-RLK genes. Developmental and stress responsive specific elements in the promoter of SD-RLK genes are shown by boxes of various colors

To understand the transcriptional regulation of 12 selected SD-RLK genes under abiotic stresses, we analyzed the presence of CREs in upstream promoter regions of these genes (Fig. 4). The presence of potential CREs involved in gene expression regulation under various stress-related stimuli such as anaerobic induction (ARE), cold responsiveness (LTR), dehydration responsiveness (Dehydrin, MYB, DRE1), light responsiveness (AE-box, ATCT-motif, Box 4, chs-CMA1a, GATA-motif, G-box, GT1-motif, LAMP-element, MRE), oxidative stress responsive (as-1), stress responsive element (STRE), wound responsiveness (WRE3, WUN-motif) were found. Additionally, development associated CREs such as light response and C-metabolism (as-1), endosperm expression (GCN4_motif), floral homeotic gene expression (F-box) also noted. A wide range of plant growth regulator responsive elements such as auxin responsiveness (TGA-element), GA3 responsiveness (GARE-motif), ABA responsiveness (ABRE, ABRE3a, ABRE4), MeJA responsiveness (CGTCA-motif, TGACG-motif), salicylic acid responsiveness (W box), and ethylene responsiveness (ERE) were also found. Thus, the computational prediction of CREs suggested that the transcription of SD-RLK genes is regulated by multiple abiotic stresses in addition to plant growth, development, and hormone perception.

### *A. thaliana* seedlings experienced oxidative stress after being exposed to diverse abiotic stresses

**Fig. 5:**
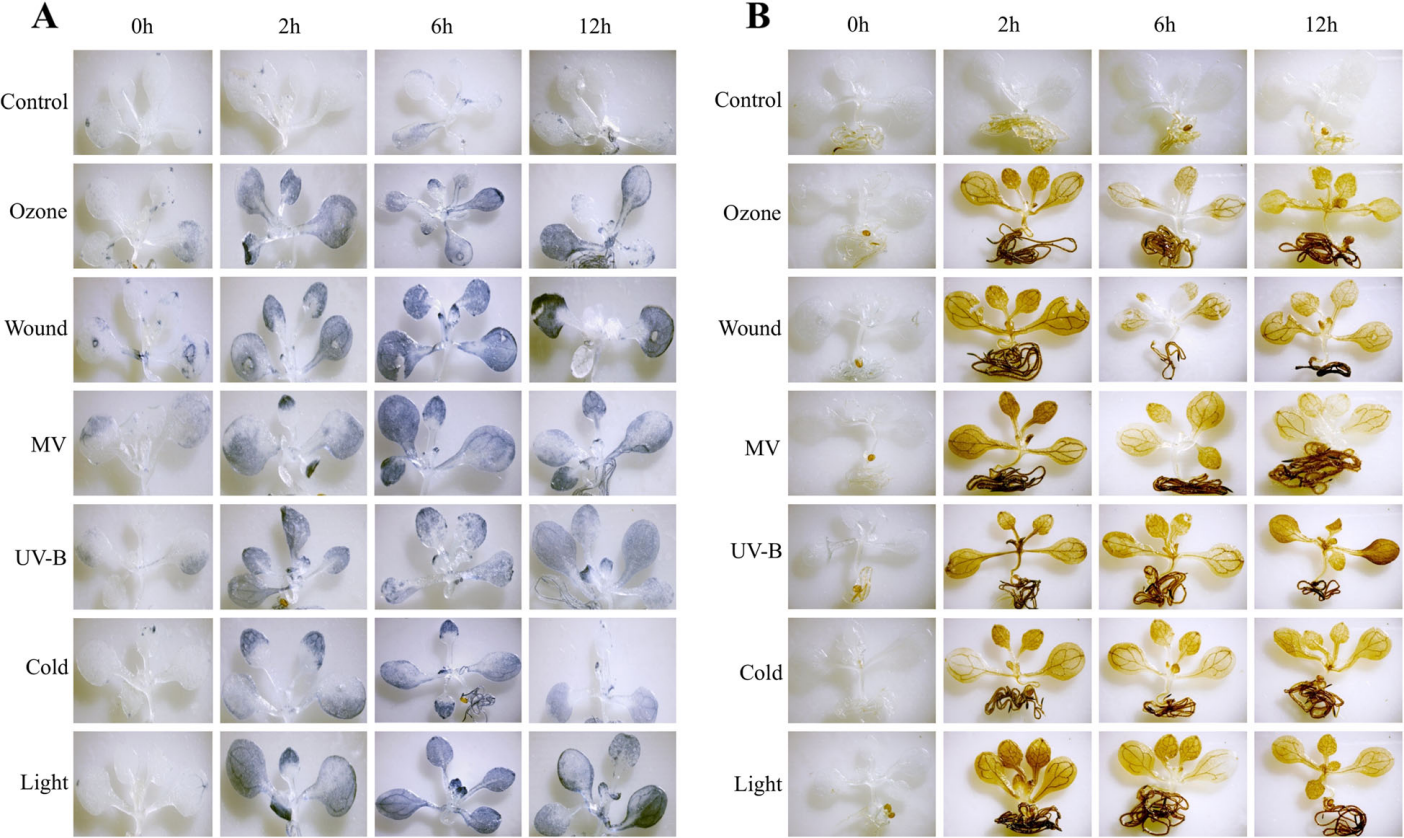
Visualization of two classic stress markers i.e. superoxide radical and hydrogen peroxide detected by NBT (**A**) and DAB (**B**) staining based methods, respectively. Detections have been done on 10 days old MS-grown *A. thaliana* seedlings subjected to different abiotic stresses after 0, 2, 6 and 12 h treatments. Bar = 100px

In 10-days-old seedlings of *A. thaliana*, two classical oxidative stress markers i.e. superoxide radical and hydrogen peroxide were measured after 0, 2, 6, and 12 h exposure to tested abiotic stresses (Fig. 5). As per our expectation, the unstressed seedlings (control) remained colourless in both staining procedures. Throughout the experiment, stressed seedlings had greater levels of superoxide radical and hydrogen peroxide than control seedlings. In 0 h of stress treatments, superoxide radicals were generated only in wounded plants. All of the stressors caused the production of superoxide radical and hydrogen peroxide within the first 2 h of treatment, and the production of them continued both in 6 h and 12 h time points. The images (Fig. 5 A and B) depict the differential accumulation of superoxide radical and hydrogen peroxide in different stresses at different time points after exposure.

### Semi-qRT-PCR expressional analysis validated the differential expression of 12 selected SD-RLK genes against abiotic stresses

From microarray-based expression profiling (Fig. 3), 12 promising differentially expressed SD-RLK genes were selected. To investigate their relative temporal transcript expression pattern, six abiotic stresses such as ozone, wounding, oxidative (MV), UV-B, cold, and light stress were selected and semi-qRT-PCR was employed. All 12 selected genes, i.e. At4g27300, At1g61440, At4g21390, At1g61610, At1g11330, At1g61380, At1g61460, At1g61430, At4g21380, At1g61360, At1g61480, and At1g61420, showed significant upregualted or downregulated expression across the tested stresses at 0 (control), 2, 6 and 12 h of exposure (Additional file 4: Fig. S2). Close evaluation of the results revealed that four genes, namely At1g61380, At4g27300, At1g61460, and At1g61360, had significantly increased/decreased expression levels in the majority of the studied stress conditions, which were consistent with the exposed time points.

### The four selected SD-RLKs behaved as potential key representative abiotic stress-responsive genes

**Fig. 6:**
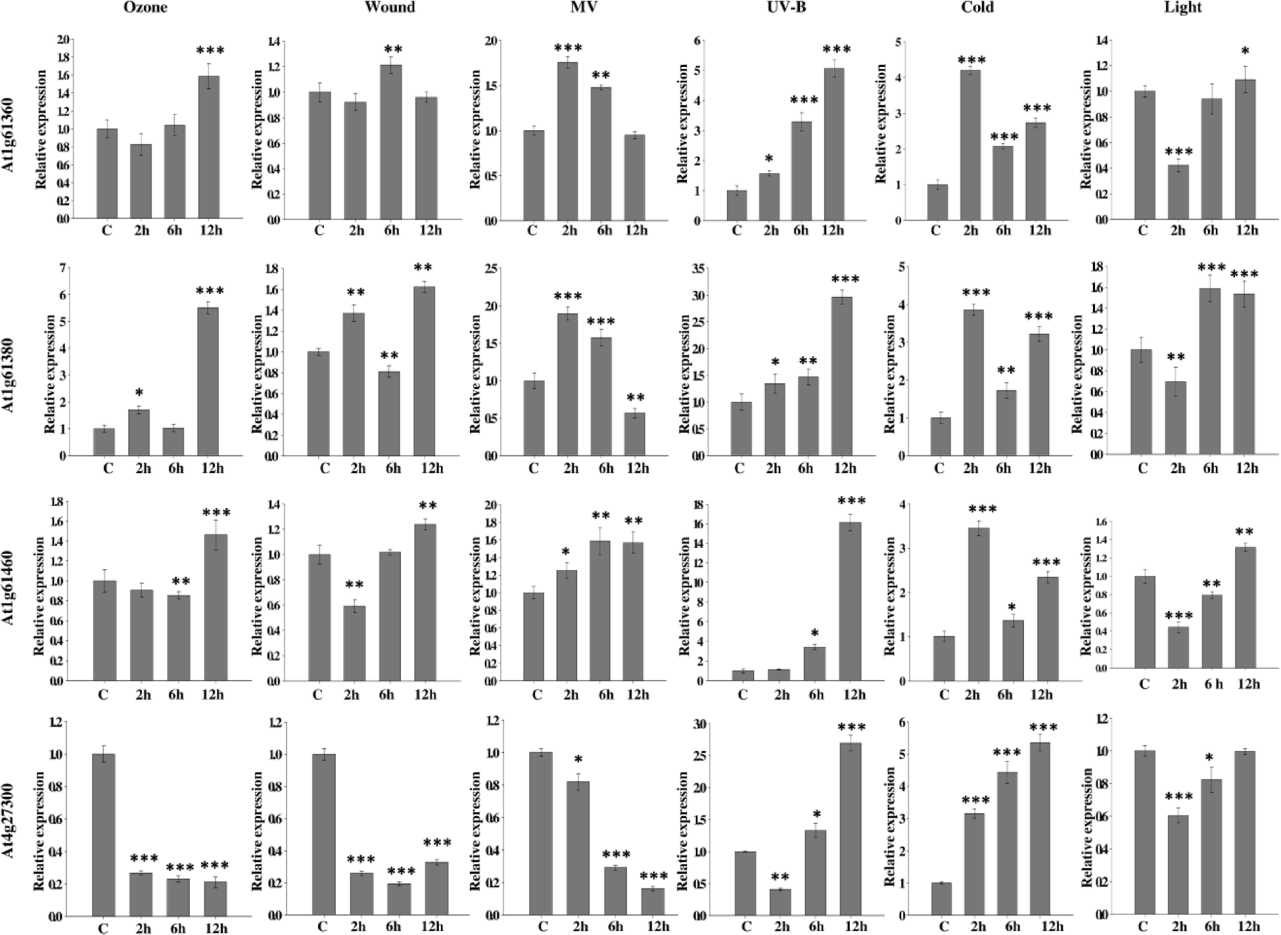
Quantitative real time-PCR (qRT-PCR) of four key representative *A. thaliana* SD-RLK genes in response to six selected abiotic stresses. Selected stress conditions are ozone (40 ppb), wounding, methyl viologen (25 μM), cold temperature (8 °C), UV-B (0.99 w m^− 2^ s^− 1^) light (500 μmol quanta m^− 2^ s^− 1^) for 2, 6, and 12 h. Relative changes in mRNA level were normalized with two reference genes, i.e. *AtAPT* and *AtEF1α*. Data represents the mean of fold increase over control sample ± SD of three biological replicates (*n* = 3). A single asterisk (*) indicates *p* < 0.05, a double asterisk (**) indicates *p* < 0.01, and a triple asterisk (***) indicates *p* < 0.001 for one–way analysis of variance (ANOVA), followed by Tukey’s post hoc test between control and treated samples

The identified four candidate SD-RLK genes showing relatively higher expression levels in semi-qRT-PCR analysis, were selected for qRT-PCR analysis (Fig. 6). The expression analysis of At1g61360 revealed a significant increase after exposure to ozone, MV, UV-B and cold stress at 2, 6, and 12 h. In light stress, its expression decreased after 2 h of exposure and stabilized at 6 and 12 h compared to control. In response to ozone and UV-B stress, maximum increase in the transcripts level of At1g61360 was observed at 12 h of exposure, and it was 1.58 and 5.60 fold, respectively. In MV and cold treatments, maximum expression was observed after 2 h of exposure with 1.75 and 4.20 fold increase as compared with control, respectively. However, no significant change in the transcript level of At1g61360 till 12 h was observed in response to wounding. Interestingly, At1g61360 demonstrated significant up-regulation in response to UV-B, followed by cold treatment, compared to any other investigated stress.

The expression pattern of At1g61380 followed nearly the same trend as At1g61360 in most of the analysed stress treatments. Significant increase in its transcript was observed after the 12 h of exposure to ozone, wounding, UV-B, and light stress with the fold change of 5.50, 1.62, 2.96 and 1.53, respectively. In response to MV treatment, an increase in its expression was observed after 2 and 6 h, followed by a decline of 0.56 fold after 12 h. In response to both MV and cold exposure, the maximum increase in its expression was observed after 2 h with a fold change of 1.85 and 3.80, respectively.

For At1g61460 expression, the maximum up-regulation of 1.46, 1.25, 1.56, 16.13, and 1.31 fold was noted after 12 h exposure to ozone, wounding, MV, UV-B, and light, respectively. In cold stress, the maximum increase of 3.2 fold in its transcript level was observed after 2 h of treatment; however, its level was maintained up to 2.3 fold till 12 h of exposure compared to control. Amongst all treatments, At1g61460 showed the most expression under UV-B stress, followed by cold stress.

Unlike At1g61360, At1g61380, and At1g61460, the At4g27300 gene showed most distinct expression pattern under the tested abiotic stresses. In response to ozone, wounding, MV and light stress treatments, the gene was significantly down-regulated with 0.21, 0.32, and 0.16 fold decreased expression, respectively. However in light stress, the relative expression level of At4g27300 was initially down-regulated 0.60 fold after 2 h compared with control; subsequently, its expression level was increased to basal level at 12 h. Nevertheless, it had more or less a similar pattern of expression to other three SD-RLK genes observed in response to UV-B and cold stress as its expression was reported to gradually increase with increase in exposure duration. The transcript level of At4g27300 was shown to be highest after 12 h of exposure to UV-B and cold, with 2.69 and 5.50 fold increases, respectively, as compared to the control.

Our results demonstrate that all four genes were significantly up-regulated in response to UV-B and cold stress. Moreover, we observed that the genes, namely, At1g61360, At1g61380, and At1g61460, showed mostly up-regulated expression; however, the expression of At4g27300 was either up- or down-regulated across the studied stress. Thus, qRT-PCR data analysis authenticates their possible involvement in abiotic stress tolerance and related signaling.

## Discussion

The transcriptional regulation involved in specific alterations in gene expression is an important and well-studied aspect of the response to a variety of abiotic stresses [28]. Because the role of 37 SD-RLKs in response to various abiotic stresses is unexplored, we investigated the structural and functional aspect of SD-RLKs genes in terms of chromosomal localization, evolutionary relationship, sequence analysis and transcript dynamics under various abiotic stresses.

Most plant gene families originated through gene duplication events [55]. In many cases, a number of gene families evolved through the duplication of whole genomes or the duplication of the individual chromosome. The other possible mechanisms of gene duplication are tandem duplication, segmental duplication, transposon-mediated gene duplication and retro-duplication [56, 57]. In our case, the phylogenetic analysis and chromosomal distribution patterns of SD-RLK genes indicated that the expansion of this gene family in *A. thaliana* may have occurred through multiple tandem gene duplications on chromosomes 1 and 4. Of all the duplicated genes in *A. thaliana*, ~ 75% contain only two genes or rarely three genes [58]. However, we found more than four tandem duplicated gene clusters on chromosomes 1 and 4, and the number of duplicated genes in these clusters varied from two to 11, indicating that numerous tandem duplication events may have had a role in the divergence of SD-RLK genes in *A. thaliana*. Nevertheless, such gene duplication benefits the acclimation of plants in more adverse environmental conditions, including abiotic stress tolerance. Since, most of the abiotic stresses are known to enhance the ROS level in different intra and extracellular compartments, thus there is a possibility that cysteine-rich ectodomain of SD-RLKs act as redox sensor and subsequently mediate the abiotic stress mitigation and related signaling in *A. thaliana*.

Further, to examine the possible function of 37 SD-RLK genes under abiotic stress conditions, we analyzed their expression profiling based on the publicly available microarray data across a wide range of abiotic stresses, namely, cold, osmotic, salt, drought, genotoxic, oxidative, UV-B, wounding and heat from both the shoot and the root tissues. The microarray data analysis suggested that out of 37 SD-RLKs genes, the expression of 12 genes were significantly modulated across all selected abiotic stresses in both root and shoot.

The CREs, mostly located upstream of the promoter sequences of a gene, and act as the potential element for transcriptional regulation by recruiting different transcription factors (TF) in response to environmental stimuli [59]. Thus, the first level of gene regulation is determined by CREs. Hence, a complete understanding of the transcriptional gene regulation system will rely on the successful functional analyses of CREs. The computational prediction of the promoter sequences of 12 selected SD-RLKs demonstrated the presence of the various CREs related to stress and developments (as mentioned in result section) that involved plant growth under stress conditions. This suggested that gene expression of SD-RLKs may be controlled by various environmental signals such as light, cold, wounding, and dehydration [59]. Although the computational identification of particular DNA sequence motifs may not demonstrate a functionally active site, the presence of these CREs provides a beforehand indication regarding the nature of the stress signal that could modulate the expression of these genes.

In the light of above observation, we subjected *A. thaliana* seedlings to the six abiotic stresses and performed semi-qRT-PCR expression analyses for 12 target genes of SD-RLK at 2, 6, and 12 h of stress exposure. Out of 12 SD-RLKs, expressions of eight genes were inconsistent; therefore they were not considered for further investigation. These types of inconsistency have been reported earlier in the CRK/DUF26 subfamily of RLKs in response to ozone and plant hormone in *A. thaliana* [20]. However, the expression patterns of other four genes, namely At1g61360, At1g61460, At1g61380, and At4g27300, demonstrated consistency in expression under majority of the studied abiotic stressors and were designated key representative genes based on semi-qRT-PCR data. To cross-verify the relative expression of these four key genes, qRT-PCR analysis was performed under similar stress conditions. The qRT-PCR results confirmed the differential expression pattern of these four genes under selected abiotic stress conditions that had been found in semi-qRT-PCR data.

In the ozone stress, except for At4g27300, other three SD-RLK genes, i.e. At1g61360, At1g61460, and At1g61380, showed maximum increase in their transcripts level after exposure up to 12 h. This up-regulation in their transcript till 12 h can be explained by the fact that the gaseous molecule ozone induces a burst of ROS in apoplast and can induce extensive changes in the gene expression [60]. These results agree with the earlier study where the enhanced expression of the members of CRK/DUF26 was observed under ozone stress in *A. thaliana* [20].

Wounding by herbivores, pests or mechanical injuries have been known to induce defence responses in plants. The expressional profiling of these potential genes in response to wounding revealed a maximum but modest expression of transcripts of At1g61360, At1g61460, and At1g61380 at 6 or 12 h; however, the expression of At4g27300 was primarily down-regulated from 2 to 12 h. This induction or suppression in the transcript level could be attributed as a precautionary measure taken by plants to activate the local defence response by activating the stress-responsive genes and to repair the damages against oxidative burst linked with cell wall reinforcement after wounding [61–63].

Methyl viologen (MV), commonly known as paraquat, is often used to induce oxidative stress in plants [64]. It can generate highly reactive oxygen species (ROS) within chloroplasts. In response to MV exposure, the transcript level of At1g61360 and At1g61380 significantly increased at 2 and 6 h. However, the transcript of At1g61460 gradually increased up to12h of exposure; nevertheless, the transcript level of At4g27300 significantly decreased starting from 2 to 12 h of MV exposure. This suggests that ROS generated in the chloroplast may control the expression profiles of these four candidate SD-RLK genes. Thus, oxidative stress-induced expression of SD-RLK genes strongly shows that there is a potent connection between this gene regulation and redox metabolism and homeostasis. Accordingly, it was previously reported that an ABC1 protein kinase-encoded gene, *AtACDO1*, was up-regulated by MV treatment and protected the *A. thaliana* from *oxidative stress* [65]*.*

Light stress can induce the toxic ROS in the photosynthetic electron transport as undesired by-products and subsequently can trigger the photo-oxidative stress [66]. We observed the maximum increase in the transcript level of At1g61360, At1g61460, and At1g61380 after 12 h of exposure; however, maximum increase in the transcript level of At4g27300 was observed after 6 h of exposure. This finding implies that these four candidate genes respond positively to oxidative stress. Previous studies have found that differences in light intensities can affect the expression profile of certain RLKs in *A. thaliana*, which is consistent with our findings [20].

UV-B radiation is a well-known stressor that enhances the production of ROS and activates general stress signaling pathways in plants [67]. In this work, significant increases in relative expression levels of all four genes were observed after 12 h of UV-B exposure, indicating that they may play a role in protecting Arabidopsis against the harmful effects of UV-B generated ROS. Our results agree with the previous report that the mutant of cysteine-rich receptor-like kinase *crk5* plants displayed impaired acclimation to UV radiation [68].

Temperature is one of the most important environmental factors that affect plant growth and development. The cold tolerance of plants depends on cellular signal transduction pathways [69]. During cold stress, the three SD-RLKs, At1g61360, At1g61460, and At1g61380, exhibited increased transcript levels after 2 h of exposure and remained above control levels until 12 h, but At4g27300 exhibited maximum accumulation only after 12 h of exposure. The enhanced expressions of the genes suggest their probable roles in cold stress signaling pathways. Our observations were strengthened by presence of cold responsive CREs in their promoter regions as well as by a previous report suggesting that CRPK1 may form a complex with a cold-stimulated RLK to perceive the cold signal in plants [70].

**Fig. 7:**
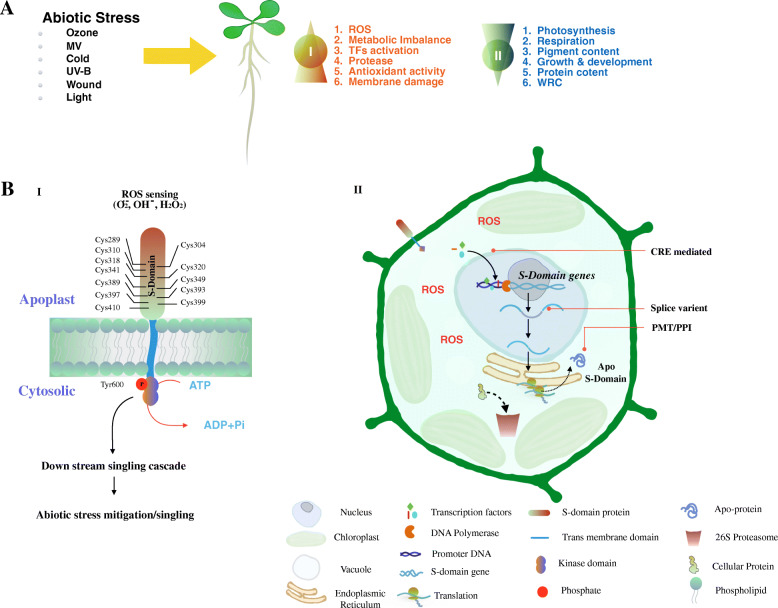
A hypothetical model showing the possible molecular mechanisms of abiotic stress mitigation and signaling in *A. thaliana* mediated by SD-RLKs. **A**. Impacts of abiotic stresses on *A. thaliana*: (I) up-regulated pathways; (II) down-regulated pathways; **B**. ROS sensing and mitigation/signaling pathways regulated by SD-RLKs (I) perception of ROS signals of SD-RLKs; (II) cascade of events led by SD-RLKs and their downstream events for mitigation/signaling events under abiotic stresses in *A. thaliana*

*The putative roles of SD-RLKs in response to abiotic stresses in A. thaliana were speculated based on above findings and summarised in the form of a hypothetical model* (Fig. *7*). During abiotic stress  Cells lose ROS homeostasis and metabolic balance in response to abiotic stressors, which increases the activity of proteases and antioxidant enzymes [71]. Furthermore, essential physiological functions like as photosynthesis, respiration, pigment content, and water retention capacity decrease, resulting in a stagnation of growth and development. In such stress conditions, the SD-RLKs may modulate the signaling pathways by sensing ROS by their conserved cysteine residues in the ectodomain. Moreover, stress-responsive TFs activate the transcription of SD-RLK genes by binding the promoter region such as MRE, STRE, W-box, CAT-box, ABRE, SARE, ERE, ARE, LTR, MYBs, and Myb. *A. thaliana* are likely to tolerate abiotic stress by altering the expression of a few key stress-responsive SD-RLK genes.

## Conclusions

Based on the results obtained, we propose that, out of 37 gene members of SD-RLKs, four key representative genes namely At1g61360, At1g61460, At1g61380, and At4g27300, play some role in abiotic stress mitigation and related signaling in *A. thaliana*. Additional research is needed to identify the precise role of these genes, as well as the pathways in which they may be involved.

## Supplementary Information

**Additional file 1.** Table S1 The total number of cysteine residues, extracellular cysteine residues, predicted disulphide bond sites forming between extracellular cysteine residues, predicted interaction partners and predicted disulphide bonding scores in 38 SD-RLKs.

**Additional file 2.** Fig. S1 Sequence alignment of ectodomain-sequence analysis of SD-RLK proteins and visualization of conserved cysteine residues.

**Additional file 3.** Table S2. List of primers used for expression analysis of selected SD-RLKs.

**Additional file 4.** Figure S2. Semi-quantitative RT-PCR (semi-qRT-PCR) of 12 SD-RLK genes under ozone, wound, methyl viologen (MV), UV-B, cold, and light stress after 0 (control), 2, 6, and 12h of stress exposures.

## Data Availability

The raw data linked to semi-quantitative RT-PCR are *available* from the corresponding *author* on *request*.
