# Supplementary material for: In silico analysis and expression profiling of S-domain receptor-like kinases (SD-RLKs) under different abiotic stresses in Arabidopsis thaliana
Source: BMC Genomics. 2021 Nov 12;22:817. doi: 10.1186/s12864-021-08133-9 (PMC8590313; doi:10.1186/s12864-021-08133-9)
Supplement: Supplementary file 1 — Additional file 1. Table S1 The total number of cysteine residues, extracellular cysteine residues, predicted disulphide bond sites forming between extracellular cysteine residues, predicted interaction partners and predicted disulphide bonding scores in 38 SD-RLKs. [file 12864_2021_8133_MOESM1_ESM.pdf]

Additional file 1: Table S1 The total number of cysteine residues, extracellular cysteine residues, predicted disulphide bond sites forming between extracellular cysteine residues, predicted interaction partners and predicted disulphide bonding scores in 38 SD-RLKs.

Serial no.	AGI	Total cysteine residues	Extracellular cysteine residues	Positions of predicted disulphide Bond forming extracellular Residues		Bonding Scores
1	AT4g27300	19	13	133	403	0.96478
				292	316	0.98624
				382	403	0.98807
2	AT4g27290	17	13	99	297	0.74554
				99	384	0.99880
				291	303	0.99539
				291	313	0.99956
				291	378	0.97769
				342	382	0.99605
				342	384	0.91939
3	AT4g03230	36	25	19	95	0.99685
				19	289	0.99740
				19	454	0.98578
4	AT1g11340	20	13	290	379	0.99336
				306	377	0.99665
				306	396	0.91944
				338	379	0.90987
5	AT1g11410	19	14	97	293	0.83119
				97	380	0.99732
				287	299	0.99957
				287	309	0.96747
				293	311	0.99963
				293	332	0.98631
				293	382	0.99885
				376	399	0.99049
6	AT1g65800	19	12	315	337	0.74512
				345	384	0.99745
				345	386	0.98154
7	AT1g65790	20	13	204	333	0.99994
				204	341	0.81780
				204	376	0.99196
				204	397	0.99495
				376	397	0.99864
8	AT4g21380	18	12	296	308	0.99223
				296	318	0.99461
				302	388	0.71393
9	AT1g11300	21	14	314	337	0.84082

				314	389	0.99396
				345	387	0.98773
10	AT1g11330	24	16	49	298	0.93941
				49	397	0.98232
				49	399	0.99524
11	AT1g11350	21	15	3	287	0.99683
				13	287	0.99730
				287	311	0.91989
12	AT4g21390	19	12	305	338	0.96673
				320	338	0.91352
				322	338	0.99342
13	AT1g61610	20	15	21	393	0.89372
				26	393	0.98326
				138	342	0.91917
14	AT1g67520	11	0	-	-	-
15	AT3g16030	19	13	334	375	0.99978
				334	377	0.99977
16	AT1g61490	19	13	11	282	0.99729
				11	333	0.96696
				11	372	0.99739
				11	389	0.97720
				282	294	0.99896
				288	378	0.93338
17	AT1g61500	20	14	7	283	0.99729
				7	326	0.99702
				7	334	0.99521
				7	373	0.92879
				7	377	0.98152
				7	379	0.99723
				7	390	0.99726
				283	295	0.87015
				283	334	0.98423
				334	377	0.99764
				369	390	0.81289
18	AT1g61420	19	12	282	288	0.99959
				282	294	0.99843
				282	302	0.98522
				333	378	0.96073
				368	378	0.99469
19	AT1g61480	21	13	196	176	0.99966
				196	378	0.99799
				282	294	0.99956
				282	302	0.99709
				282	304	0.98678
				282	333	0.96517
				288	378	0.96371
				333	376	0.97215

				333	378	0.99913
20	AT1g61550	19	13	277	289	0.99959
				277	297	0.99664
				277	299	0.98723
				283	273	0.74881
				328	384	0.99574
				363	384	0.98712
				367	384	0.95192
21	AT1g61390	20	15	9	302	0.99322
				29	302	0.99690
				302	314	0.92007
				302	324	0.99534
22	AT1g61430	19	12	281	293	0.99961
				281	301	0.99890
				281	303	0.99701
				287	377	0.98060
				332	388	0.99605
				371	388	0.78800
23	AT1g61400	19	12	292	304	0.99957
				292	312	0.99882
				292	314	0.99543
				292	386	0.99629
				298	388	0.99783
24	AT1g61440	18	12	275	287	0.99953
				275	295	0.99756
				275	297	0.99357
				326	382	0.8077
				361	382	0.94309
25	AT1g61370	20	13	22	386	0.90345
				22	397	0.98355
26	AT1g11280	23	14	35	133	0.99558
				35	397	0.98463
27	AT1g61380	22	16	8	281	0.99727
				8	375	0.82644
				281	293	0.86247
				281	303	0.99451
				371	392	0.85897
				375	392	0.98638
28	AT1g61360	22	15	6	280	0.99729
				241	378	0.99976
				280	292	0.99958
				280	300	0.95608
				280	302	0.99916
				280	331	0.93889
29	At1g61460	19	12	251	270	0.97241
				257	344	0.76298
				334	355	0.99595

30	AT4g11900	24	13	4	315	0.87184
				321	411	0.98438
				368	421	0.9909
31	AT5g60900	24	15	293	405	0.95843
				303	405	0.99811
				357	405	0.99468
				401	420	0.83695
32	AT4g00340	20	11	298	321	0.95397
				298	369	0.99906
33	At2g19130	21	13	290	302	0.99878
				290	310	0.99087
				290	312	0.97972
34	AT5g24080	21	12	183	223	0.99961
				205	223	0.99931
				207	223	0.99958
				207	281	0.99897
				223	281	0.99286
				233	281	0.99336
				277	283	0.82996
35	AT4g32300	19	13	302	371	0.99559
				315	371	0.99839
				367	385	0.99758
36	At1g34300	22	12	291	308	0.99974
				291	317	0.97204
				354	399	0.99948
37	AT5g35370	22	13	293	312	0.88799
				325	380	0.87469
				372	380	0.99552
38	At2g41890	25	15	10	298	0.99967
				10	369	0.99756
				10	413	0.98617
				10	439	0.99792
				287	308	0.99925
				287	413	0.99728
				310	323	0.96299
				310	413	0.90956
				369	439	0.99714
				375	413	0.99961
				375	439	0.99933
				375	442	0.99862
