# Supplementary material for: In silico analysis and expression profiling of S-domain receptor-like kinases (SD-RLKs) under different abiotic stresses in Arabidopsis thaliana
Source: BMC Genomics. 2021 Nov 12;22:817. doi: 10.1186/s12864-021-08133-9 (PMC8590313; doi:10.1186/s12864-021-08133-9)

**Additional file 2: Fig. S1** Sequence alignment of ectodomain-sequence analysis of SD-RLK proteins and visualization of conserved cysteine residues

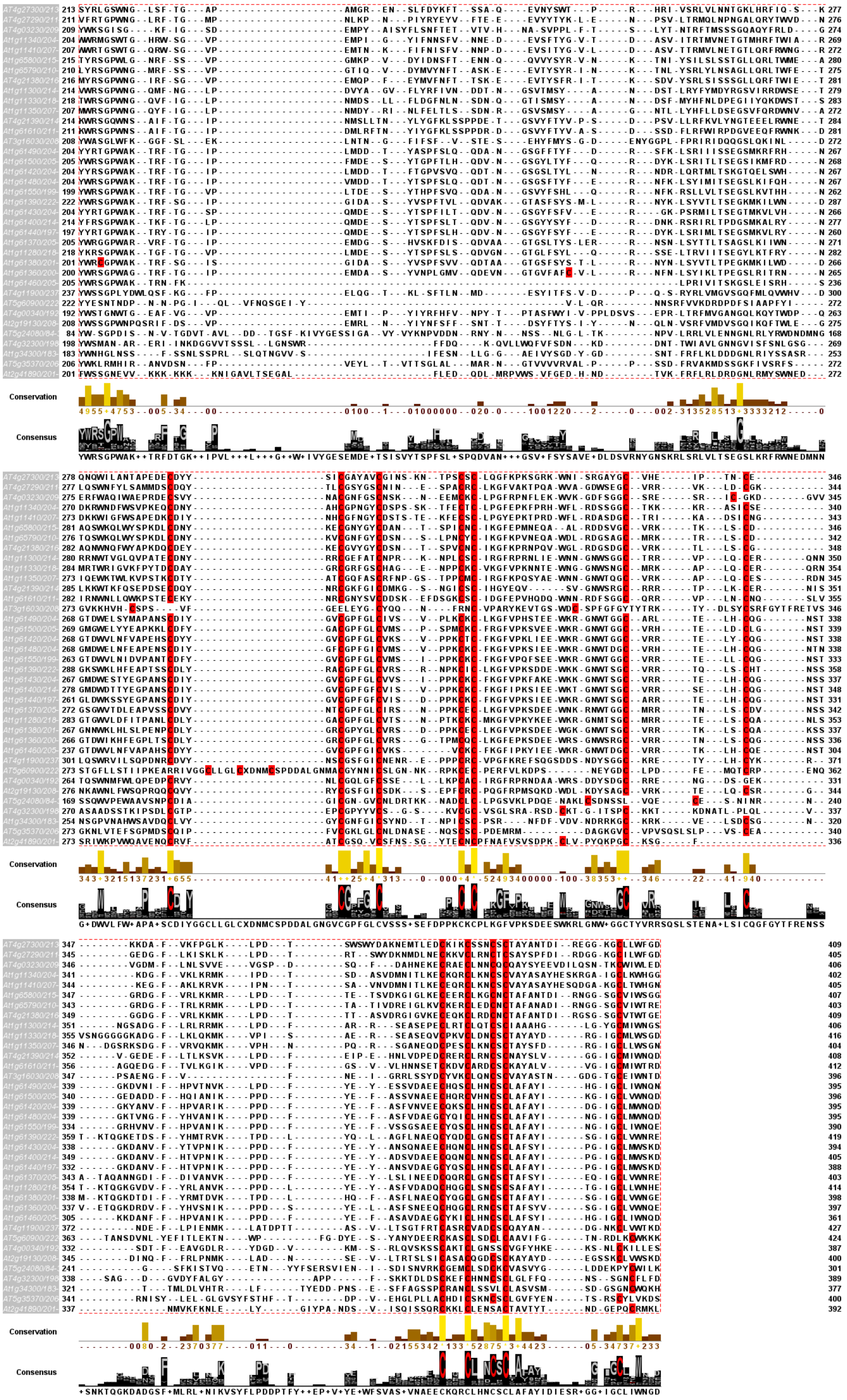

Supplement: Supplementary file 2 — Additional file 2. Fig. S1 Sequence alignment of ectodomain-sequence analysis of SD-RLK proteins and visualization of conserved cysteine residues. [file 12864_2021_8133_MOESM2_ESM.pdf]
