# Supplementary material for: In silico analysis and expression profiling of S-domain receptor-like kinases (SD-RLKs) under different abiotic stresses in Arabidopsis thaliana
Source: BMC Genomics. 2021 Nov 12;22:817. doi: 10.1186/s12864-021-08133-9 (PMC8590313; doi:10.1186/s12864-021-08133-9)
Supplement: Supplementary file 3 — Additional file 3. Table S2. List of primers used for expression analysis of selected SD-RLKs. [file 12864_2021_8133_MOESM3_ESM.pdf]

Additional file 3: Table S2. List of primers used for expression analysis of selected SD-RLKs

Serial no.	AGI	Primer name	Sequence
**1**	AT1g27450	AtAPT1 F	GAGACATTTTGCGTGGGATT
		AtAPT1 R	CGGGGATTTTAAGTGGAACA
**2**	AT3g62250	AtUBQ5 F	CCAAGCCGAAGAAGATCAAG
		AtUBQ5 R	ACTCCTTCCTCAAACGCTGA
**3**	At1g07920	AteF1α F	TGGTGACGCTGGTATGGTTA
		AteF1α R	TCCTTCTTGTCCACGCTCTT
**4**	AT4g27300	AT4g27300 F	CAGAGTCGTGGGTACTTATGGT
		AT4g27300 R	CCTCGATTCGTCTTGCCAGT
**5**	AT4g21380	AT4g21380 F	TTCTCGGTTGTGTGTGGAGG
		AT4g21380 R	CCTCTGCACGTTCTTGAACAC
**6**	AT1g11330	At1g11330 F	CTTGCAAGAATTTTCCGAGCGA
		At1g11330 R	TGACATATAGCCGTATGTTCCAA
**7**	AT4g21390	AT4g21390 F	GCGTTGTTGGAACTTACGGA
		AT4g21390 R	GCAACAAGACACCGAAGCTG
**8**	AT1g61610	At1g61610 F	AGTCGTCGGCACATATGGTT
		At1g61610 R	TCCATGATCAGTCCCTCGAA
**9**	AT1g61420	At1g61420 F	AACCCTTATTGCATATGCGTGG
		At1g61420 R	TCGGCGACATCTTTGTCCA
**10**	AT1g61480	At1g61480 F	GAACTCTAGGATATATGGCTCCCG
		At1g61480 R	CTCCCCGGTGATGATTTCCA
**11**	AT1g61430	At1g61430 F	CCTGGATACTTTTGTCTTTGATGC
		At1g61430 R	TGACCTTCAGGTCTCGGTGA
**12**	AT1g61440	At1g61440 F	ACAGCTTCGGAGTTCTTCTGT
		At1g61440 R	TCCCATACATATGCAAGAAGTGC
**13**	AT1g61380	At1g61380 F	GCATGGGCAGGATTGTTCTC
		At1g61380 R	CGCACCAAGAATCCCATGTATAA
**14**	AT1g61360	At1g61360 F	GTGTTGTAGGCACTCTAGGATACA
		At1g61360 R	TGCCAGTGATGATTTCGAGC
**15**	AT1g61460	At1g61460 F	AGGGTCGTAGGAACTCTAGGATA
		At1g61460 R	AACTCCTCCAGTTTCACACCAA
