# Supplementary material for: In silico analysis and expression profiling of S-domain receptor-like kinases (SD-RLKs) under different abiotic stresses in Arabidopsis thaliana
Source: BMC Genomics. 2021 Nov 12;22:817. doi: 10.1186/s12864-021-08133-9 (PMC8590313; doi:10.1186/s12864-021-08133-9)
Supplement: Supplementary file 4 — Additional file 4. Figure S2. Semi-quantitative RT-PCR (semi-qRT-PCR) of 12 SD-RLK genes under ozone, wound, methyl viologen (MV), UV-B, cold, and light stress after 0 (control), 2, 6, and 12h of stress exposures. [file 12864_2021_8133_MOESM4_ESM.pdf]

Additional file 4: Fig. S2 Semi-quantitative RT-PCR (semi-qRT-PCR) of 12 SD-RLK genes under ozone, wound**,** methyl viologen (MV)**,** UV-B, cold, and light stress after 0 (control), 2, 6, and 12h of stress exposures. Amplification of *AtUBQ5* and *AteFα1* were performed as an internal control in each RT-PCR reaction. One each slide the abiotic stresses are shown by red colour. For each gene, PCR product from 0 (control), 2, 6, and 12h of stress exposures, are derived from the same experiments and were loaded on same gel, with 7 wells on upper part of gel and 7 well on down part of gel, due to limitation of the gel size. The gel images of 7 upper well (including DNA ladder, control and 2h samples) and 7 lower wells (including, DNA ladder, 6h, and 12h samples) were captured separately and for comparative analysis of their transcript accumulation, these two captured images from the same experiments and same gel are aligned side by side. The edges of the each captured image were slightly cropped for better representations/alignment, however, full delineated gel images are provided here along with clearly visible loading wells. Further on each gel the red arrow shows the 100bp DNA ladder. The four key representative SD-RLKs are depicted by blue colour.

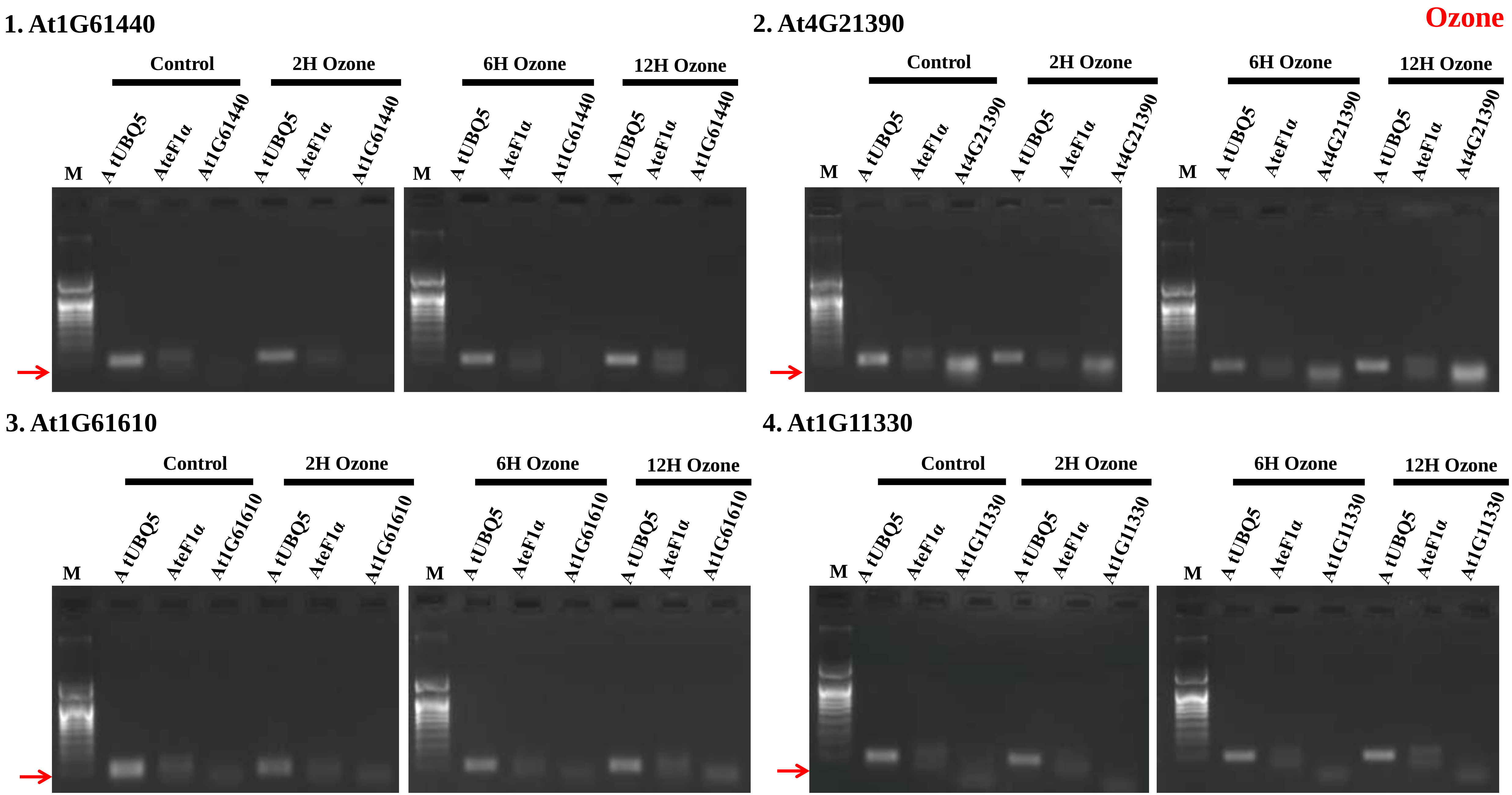
Ozone
1. At1G61440
Control
2H Ozone
6H Ozone
12H Ozone
M
A tUBQ5
AteF1α
At1G61440
2. At4G21390
Control
2H Ozone
6H Ozone
12H Ozone
M
A tUBQ5
AteF1α
At4G21390
3. At1G61610
Control
2H Ozone
6H Ozone
12H Ozone
M
A tUBQ5
AteF1α
At1G61610
4. At1G11330
Control
2H Ozone
6H Ozone
12H Ozone
M
A tUBQ5
AteF1α
At1G11330

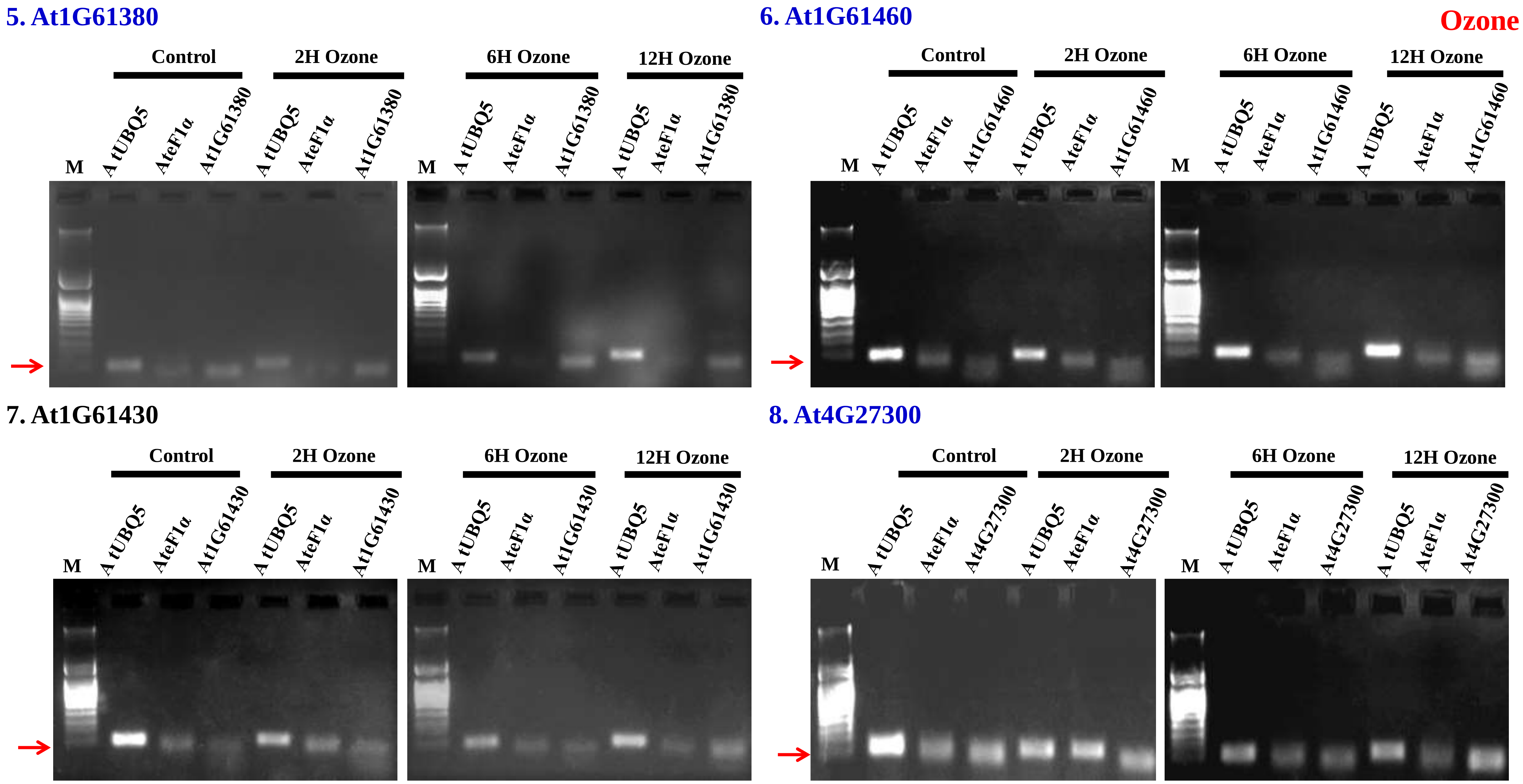
Ozone
5. At1G61380
Control
2H Ozone
6H Ozone
12H Ozone
M
A tUBQ5
AteF1α
At1G61380
6. At1G61460
Control
2H Ozone
6H Ozone
12H Ozone
M
A tUBQ5
AteF1α
At1G61460
7. At1G61430
Control
2H Ozone
6H Ozone
12H Ozone
M
A tUBQ5
AteF1α
At1G61430
8. At4G27300
Control
2H Ozone
6H Ozone
12H Ozone
M
A tUBQ5
AteF1α
At4G27300

Ozone

9. At4G21380

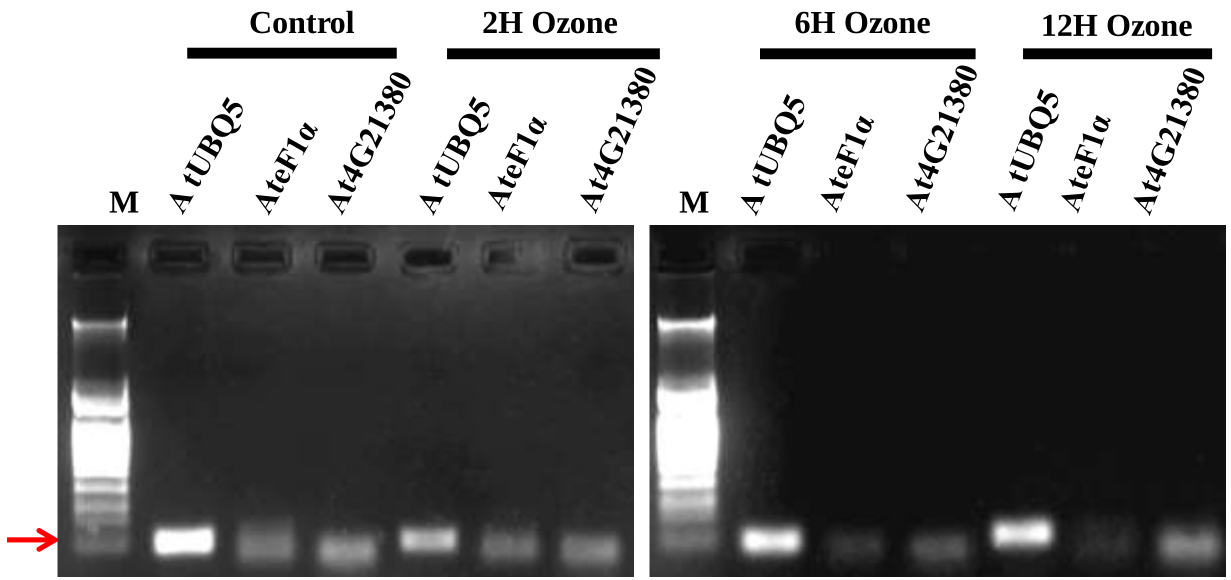

10. At1G61360

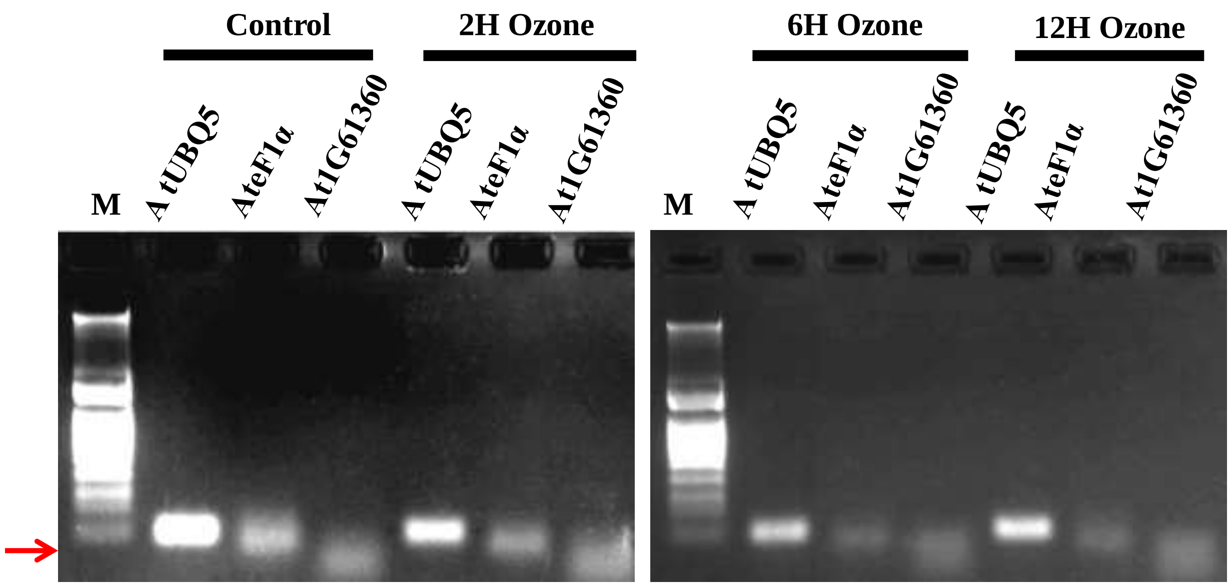

11. At1G61480

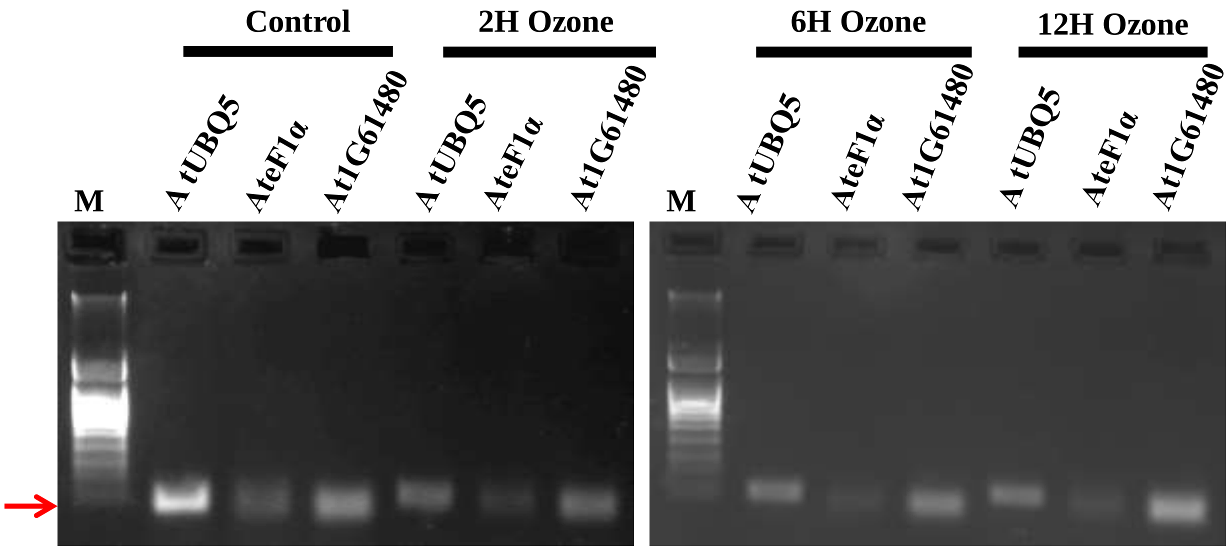

12. At1G61420

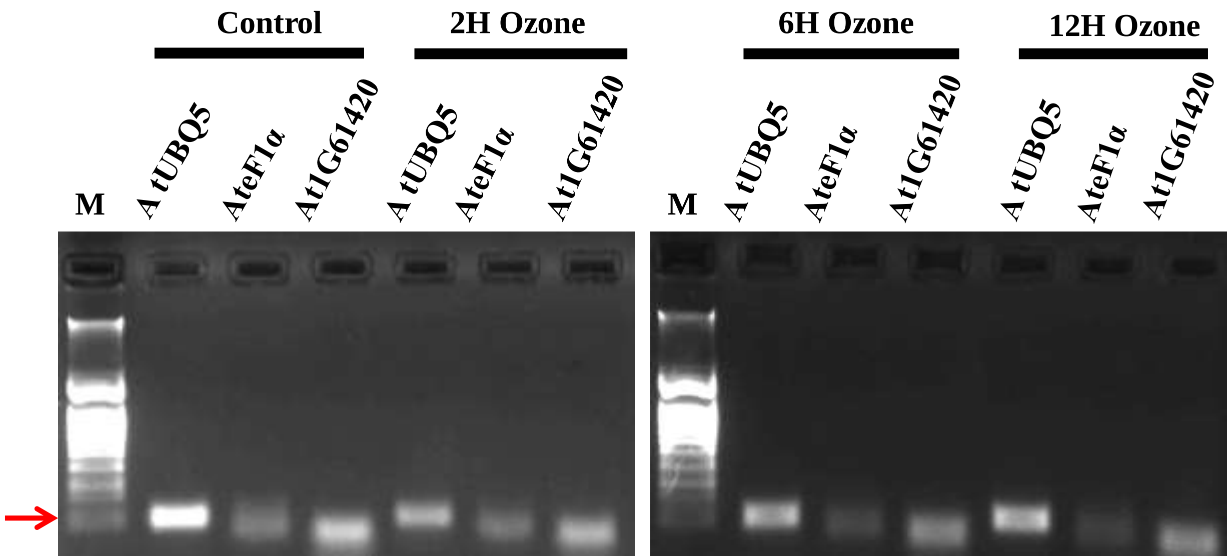

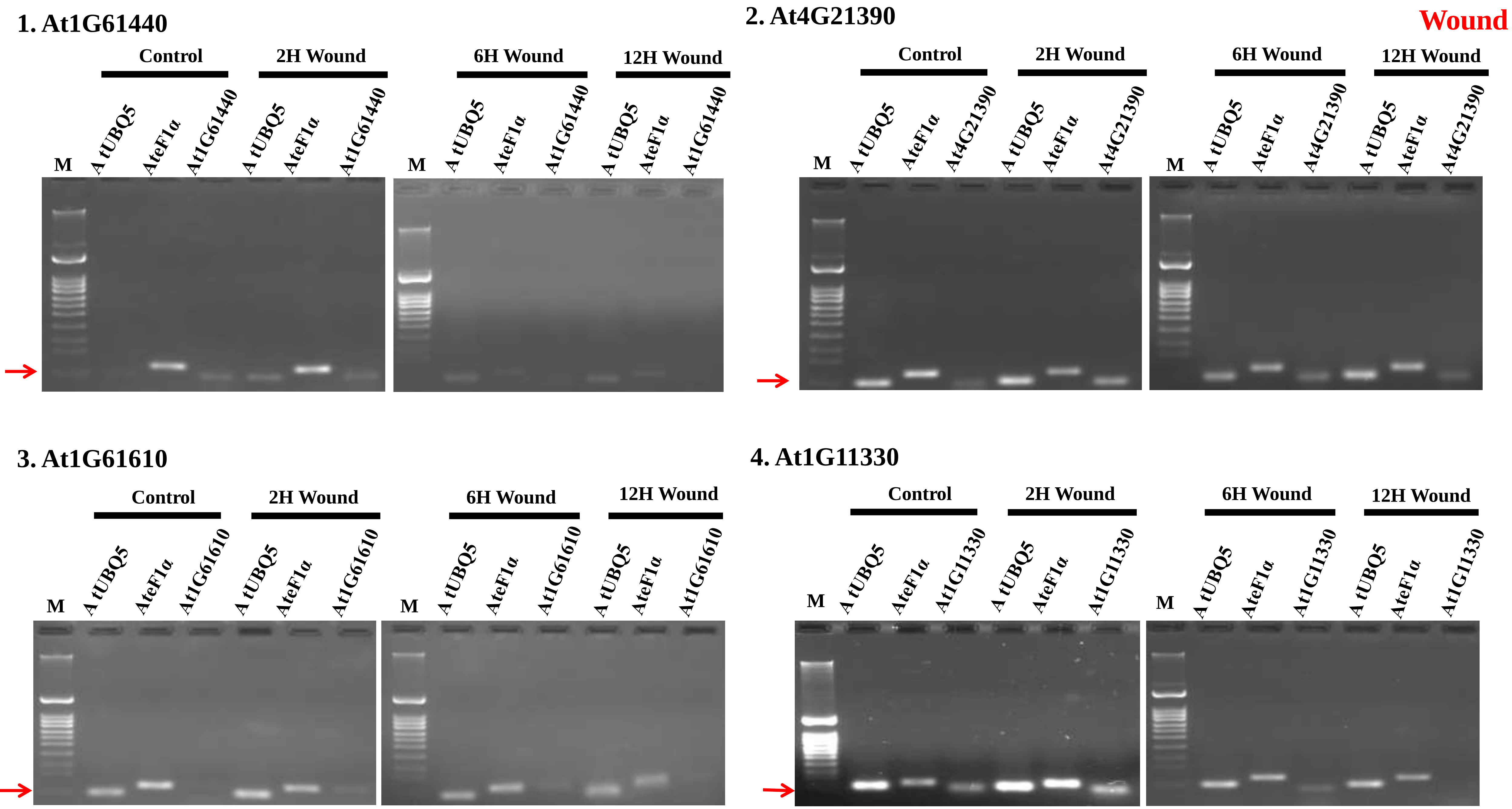

Wound
1. At1G61440
Control
2H Wound
6H Wound
12H Wound
M
A tUBQ5
AteF1α
At1G61440
2. At4G21390
Control
2H Wound
6H Wound
12H Wound
M
A tUBQ5
AteF1α
At4G21390
3. At1G61610
Control
2H Wound
6H Wound
12H Wound
M
A tUBQ5
AteF1α
At1G61610
4. At1G11330
Control
2H Wound
6H Wound
12H Wound
M
A tUBQ5
AteF1α
At1G11330

Wound

5. At1G61380

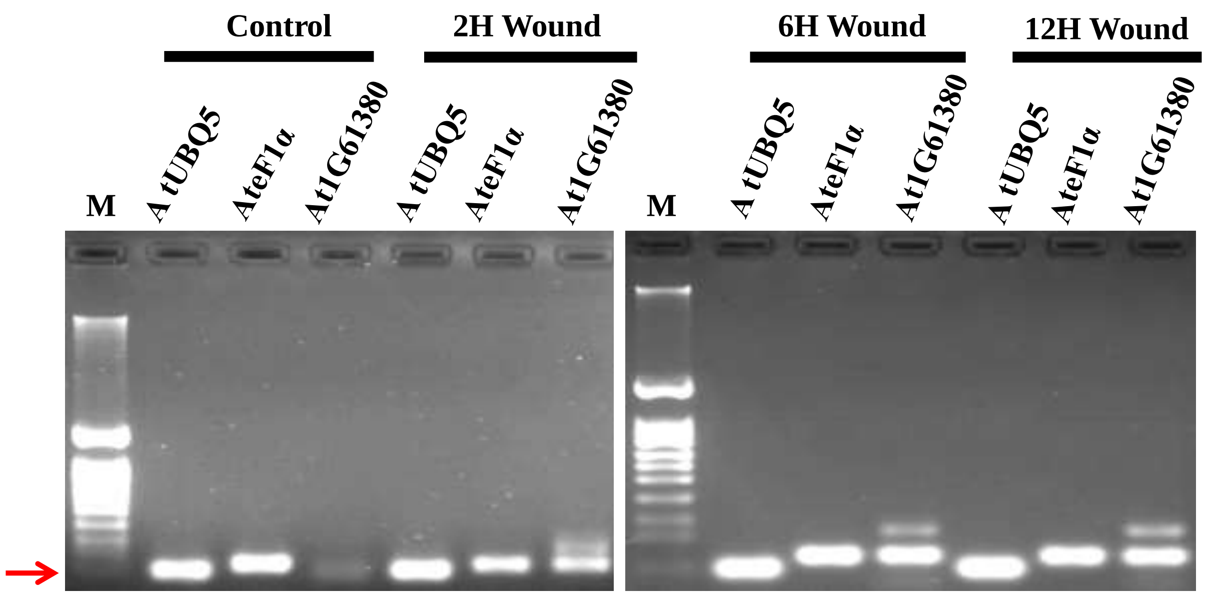

6. At1G61460

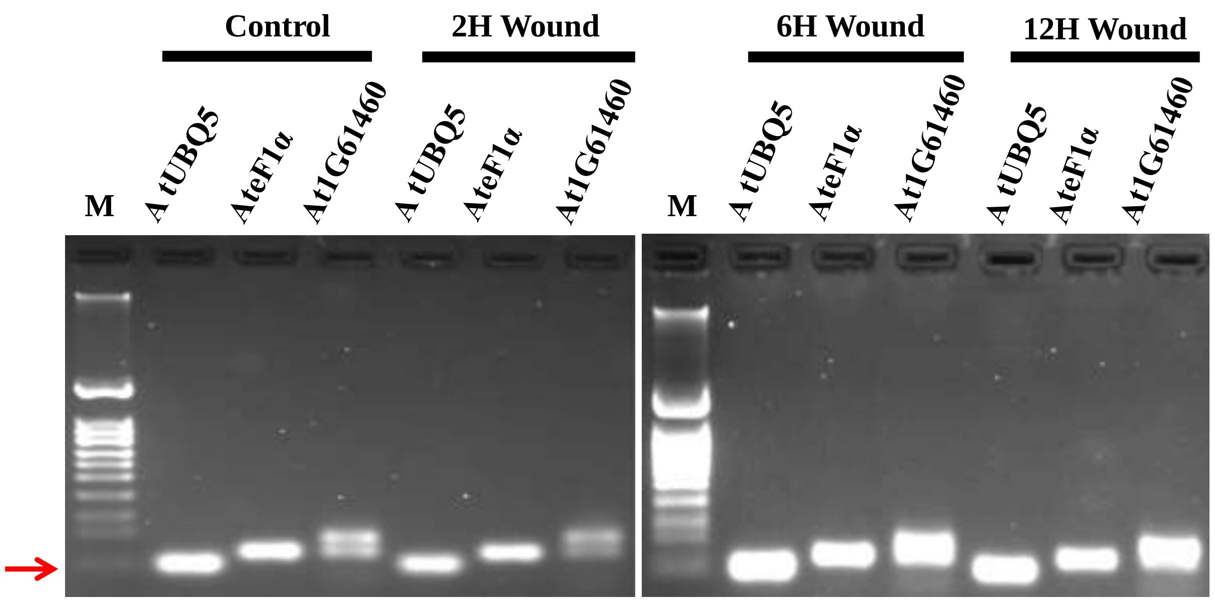

7. At1G61430

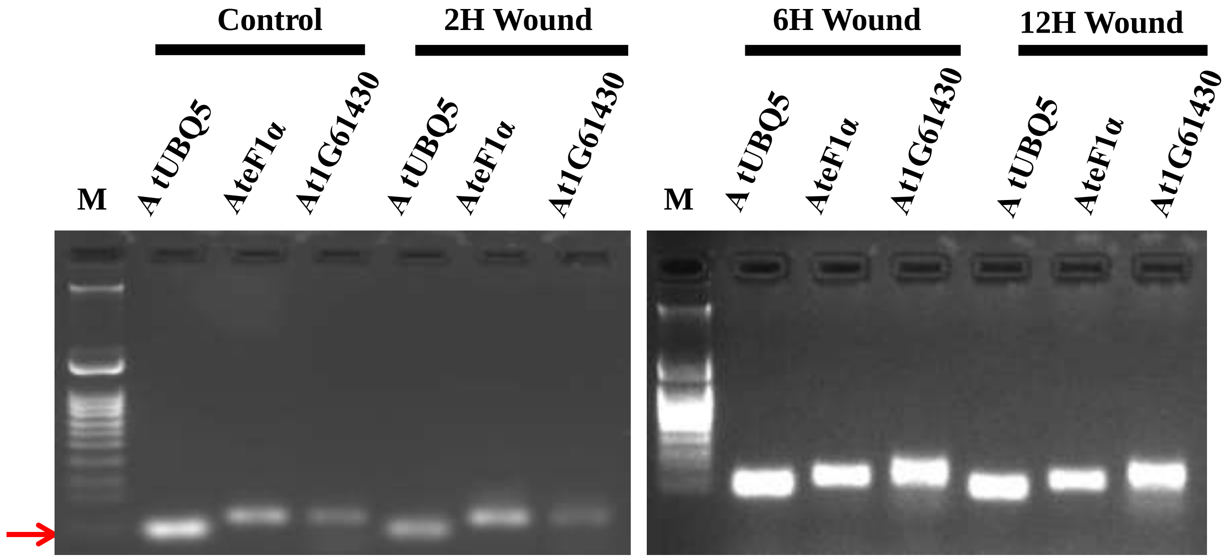

8. At4G27300

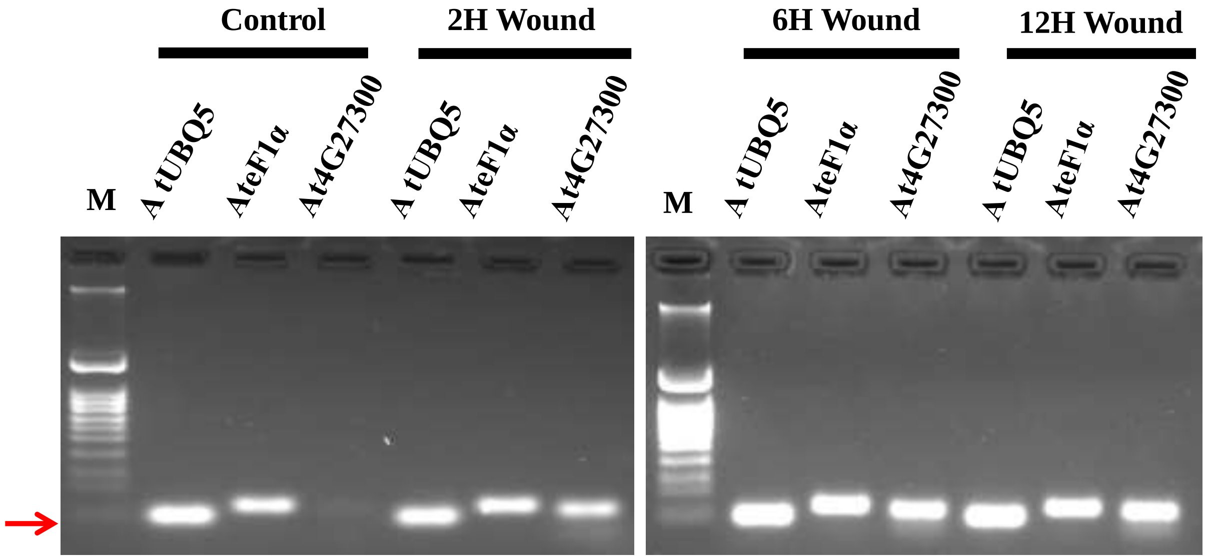

Wound

9. At4G21380

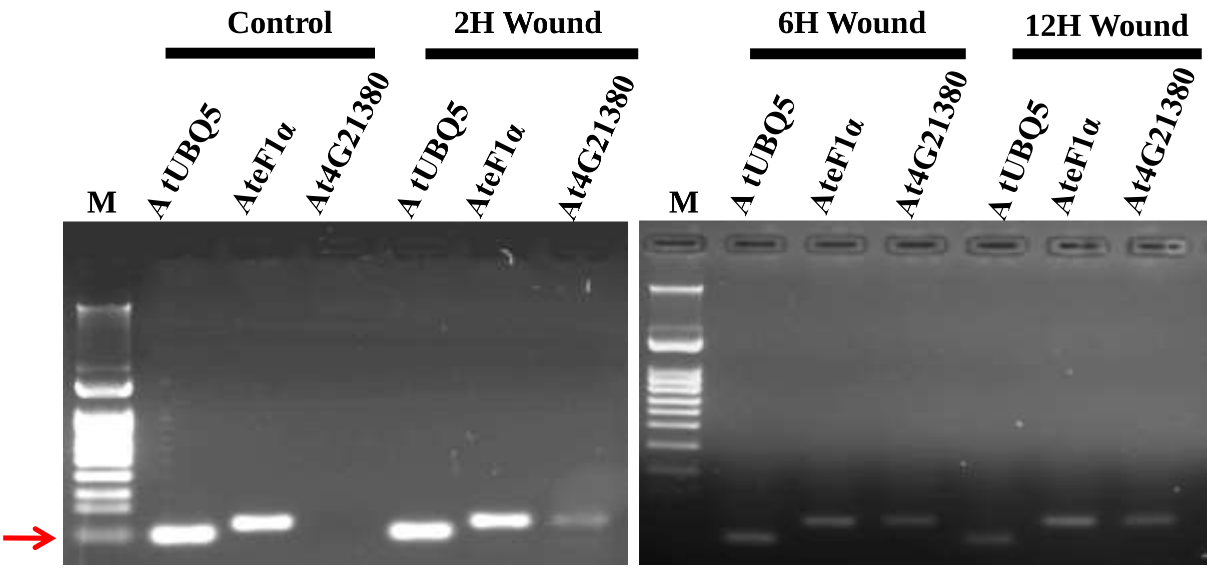

10. At1G61360

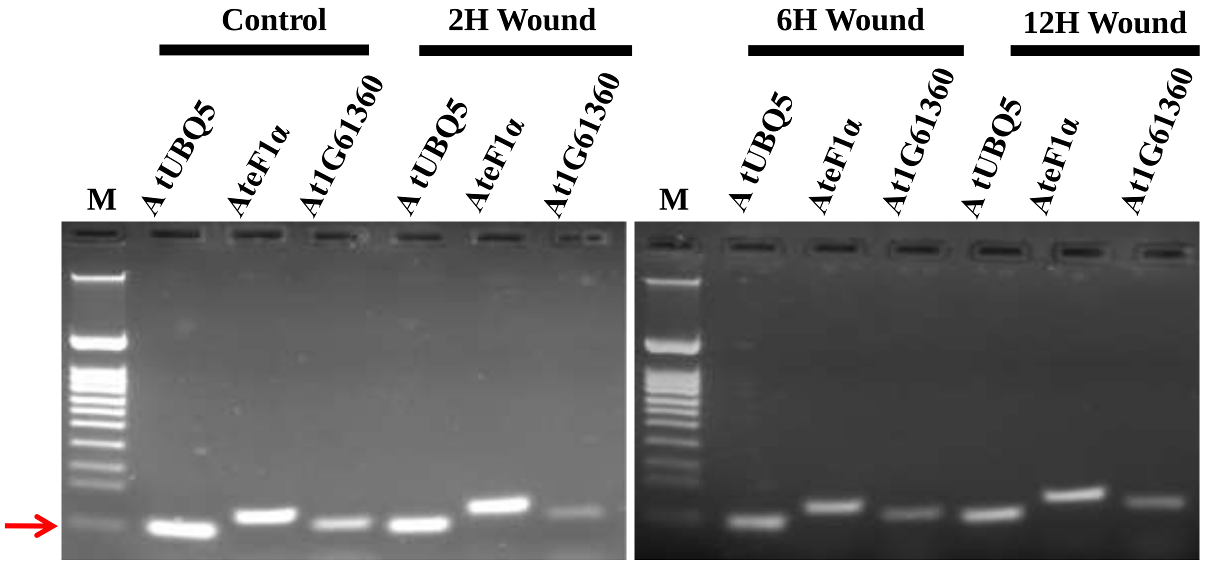

11. At1G61480

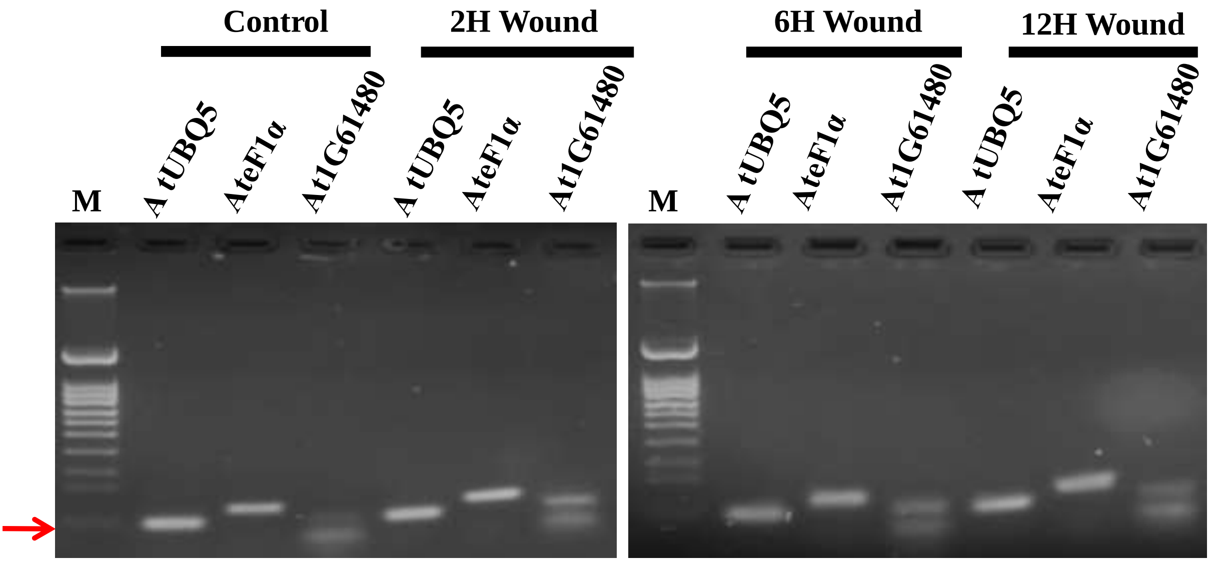

12. At1G61420

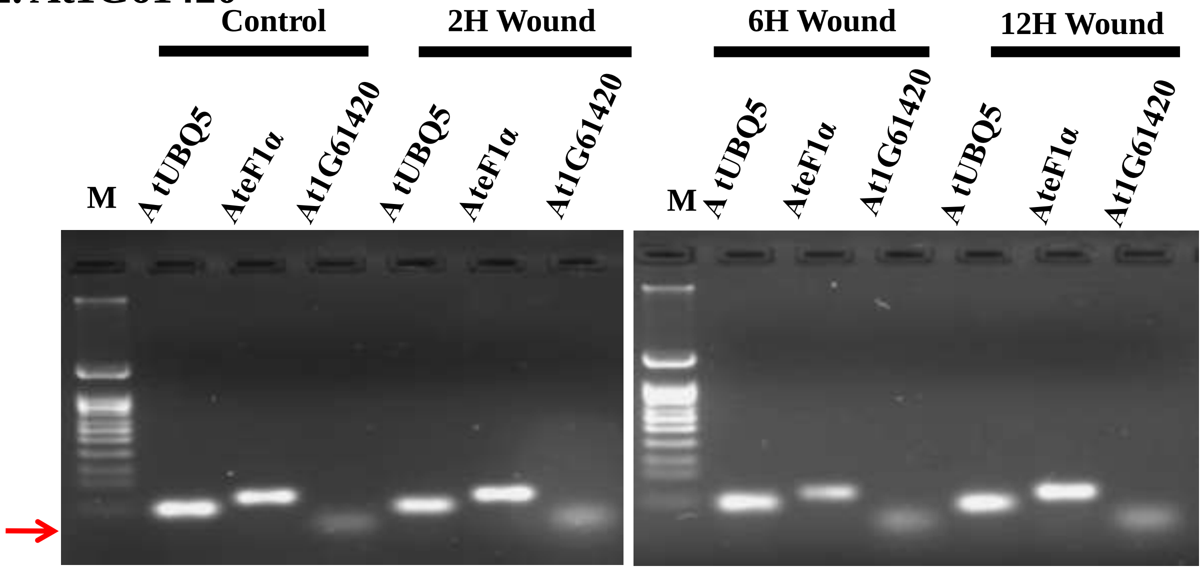

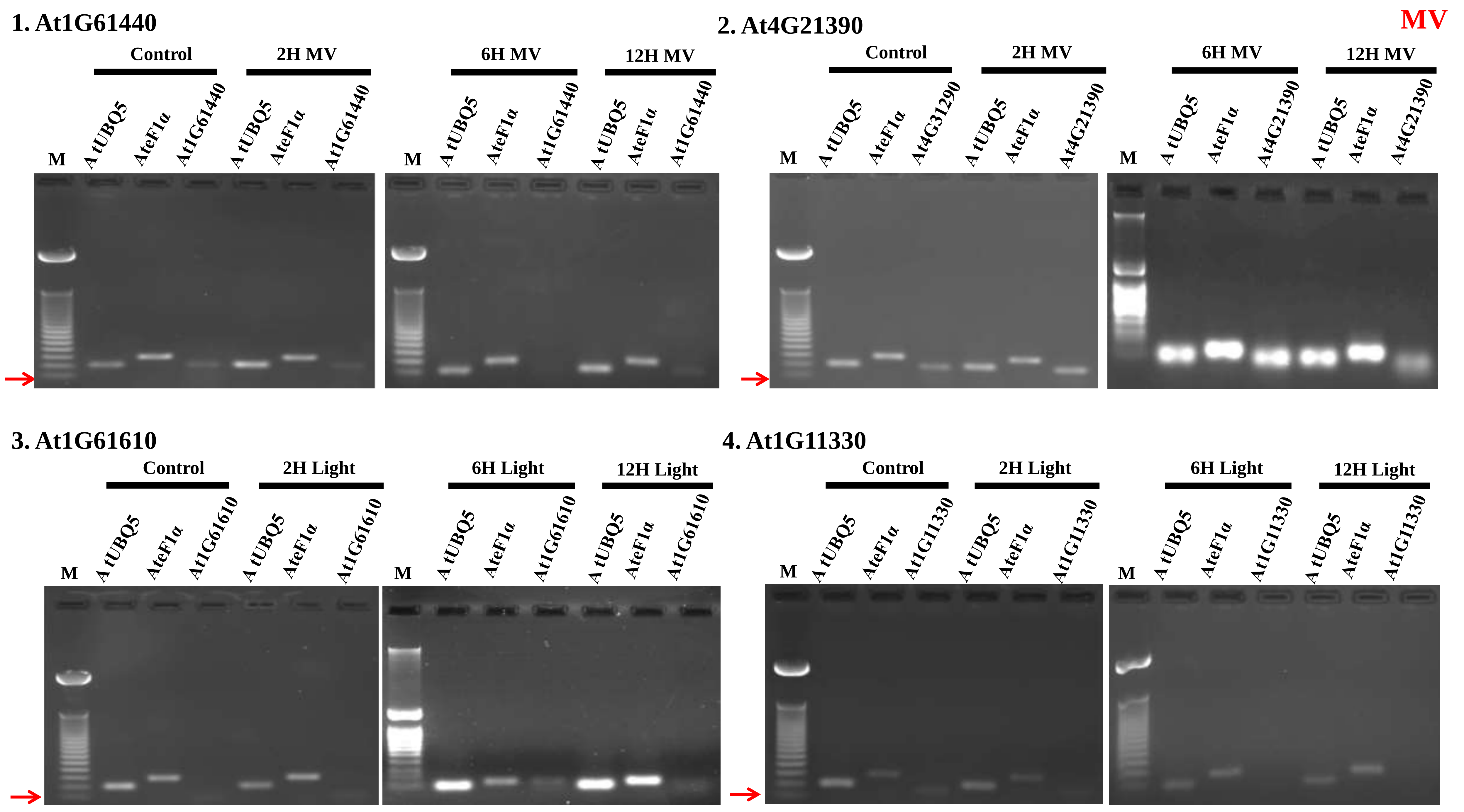

MV
1. At1G61440
Control
2H MV
6H MV
12H MV
M
A tUBQ5
AteF1α
At1G61440
2. At4G21390
Control
2H MV
6H MV
12H MV
M
A tUBQ5
AteF1α
At4G31290
At4G21390
3. At1G61610
Control
2H Light
6H Light
12H Light
M
A tUBQ5
AteF1α
At1G61610
4. At1G11330
Control
2H Light
6H Light
12H Light
M
A tUBQ5
AteF1α
At1G11330

MV

5. At1G61380

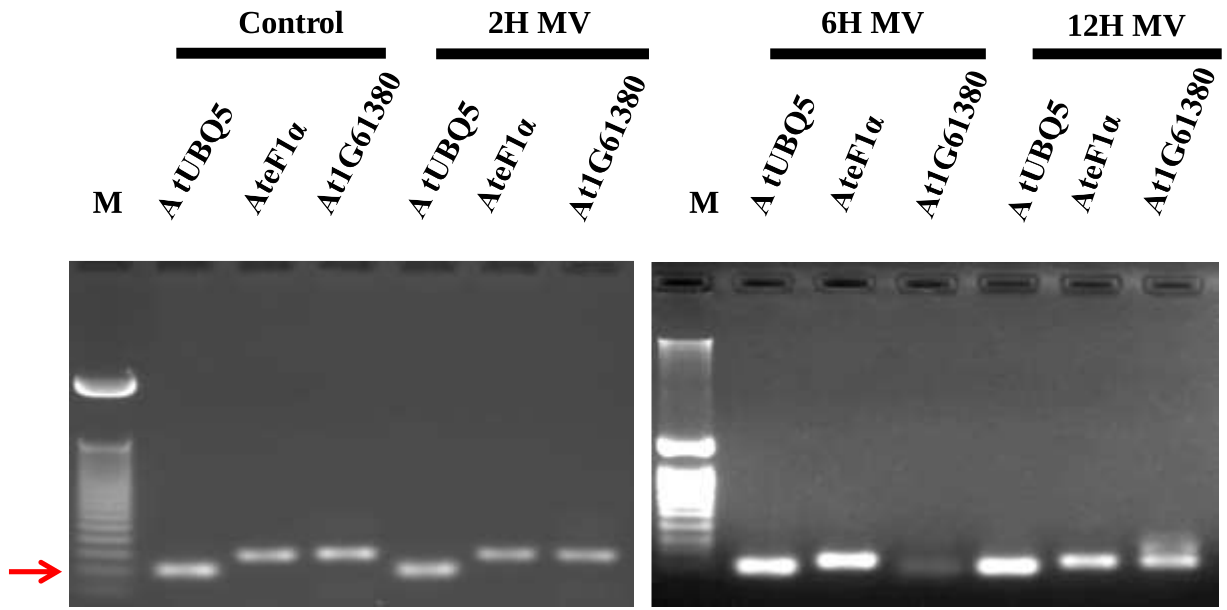

6. At1G61460

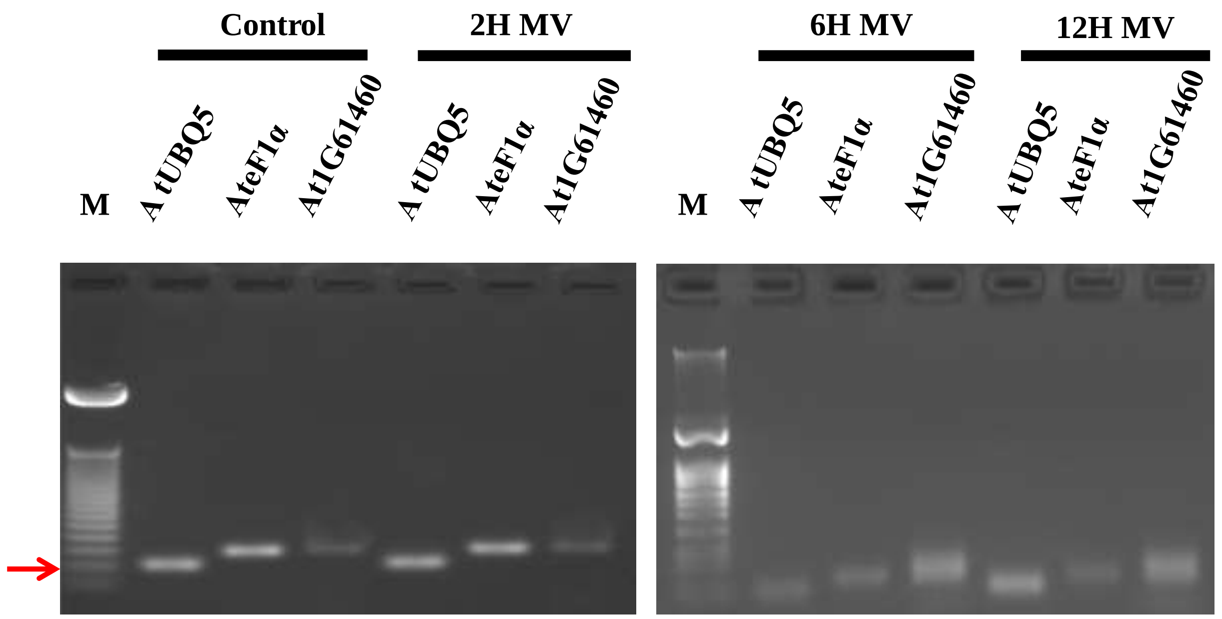

7. At1G61430

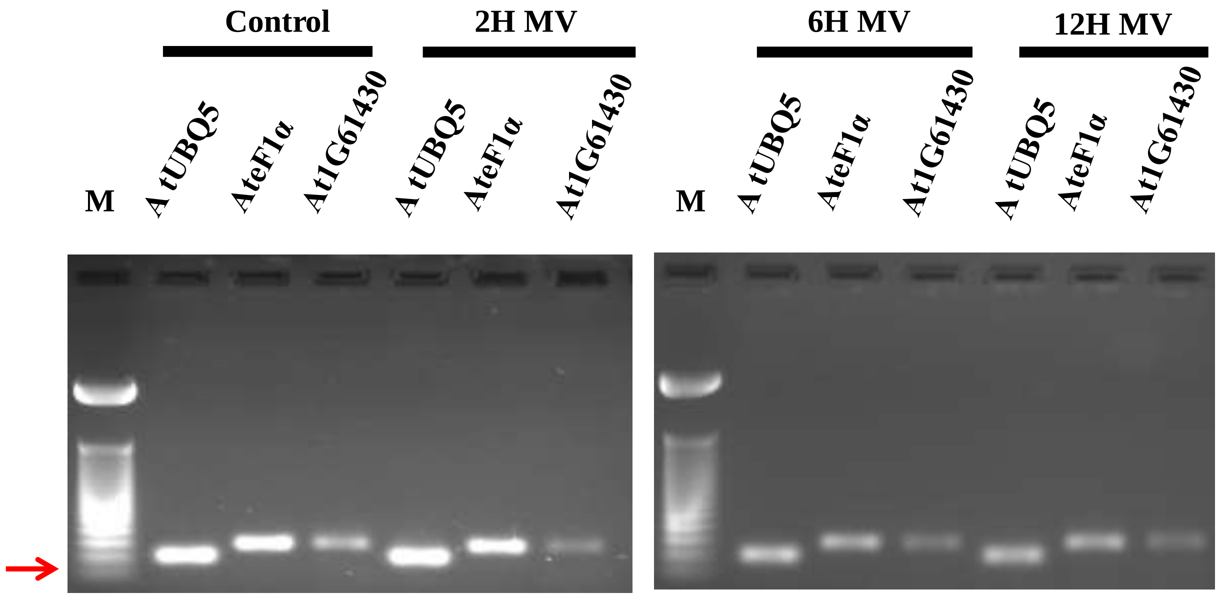

8. At4G27300

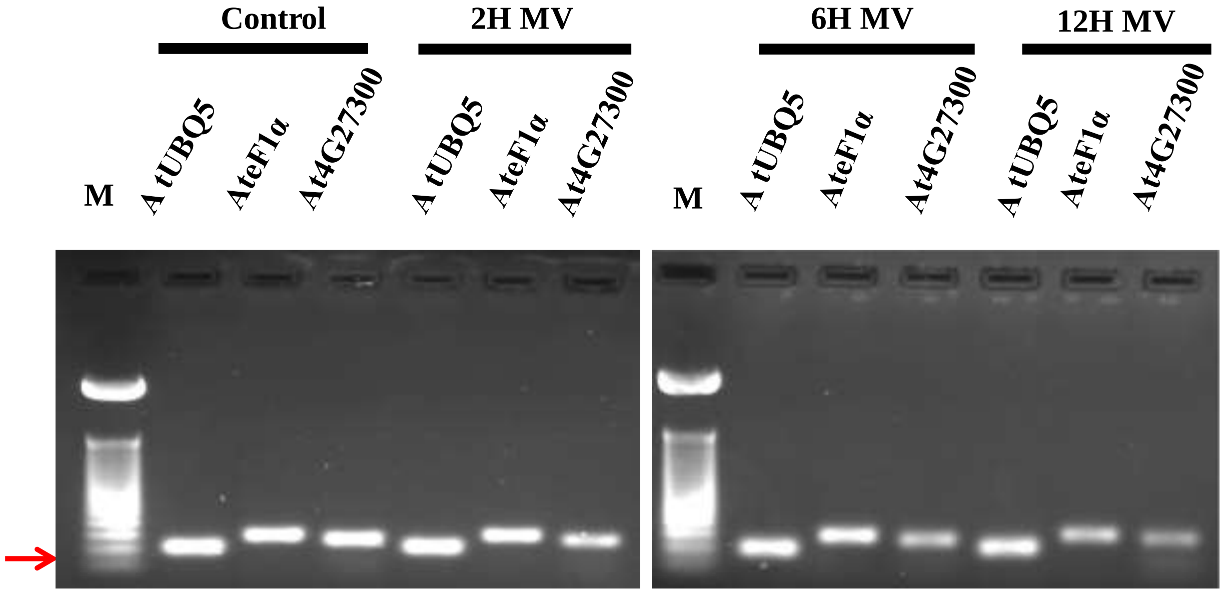

MV

9. At4G21380

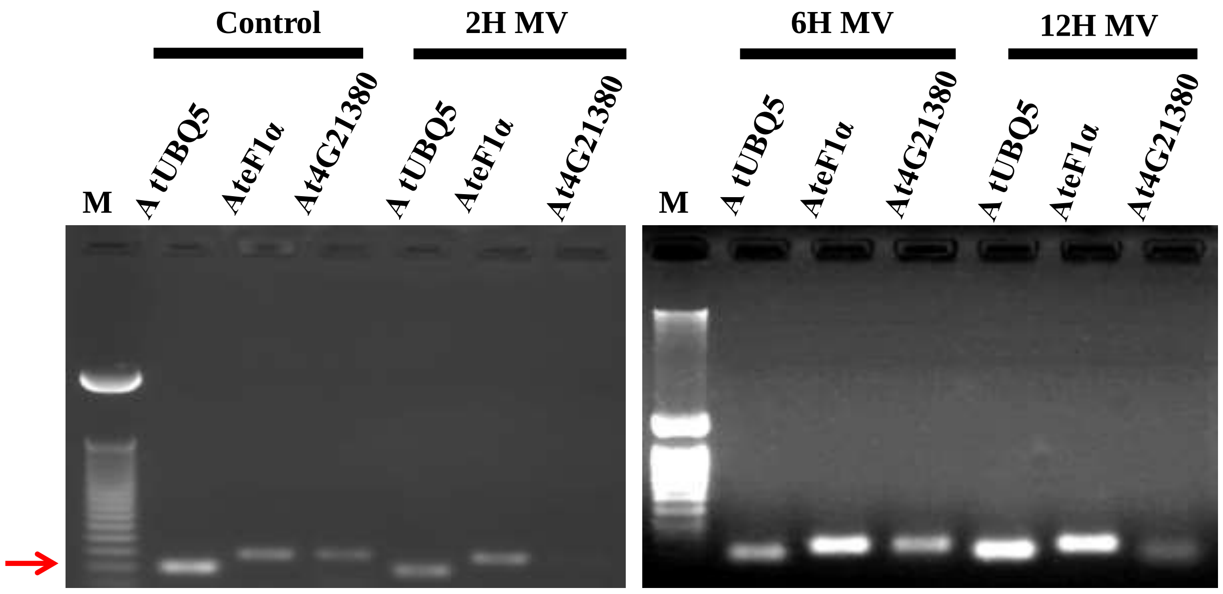

10. At1G61360

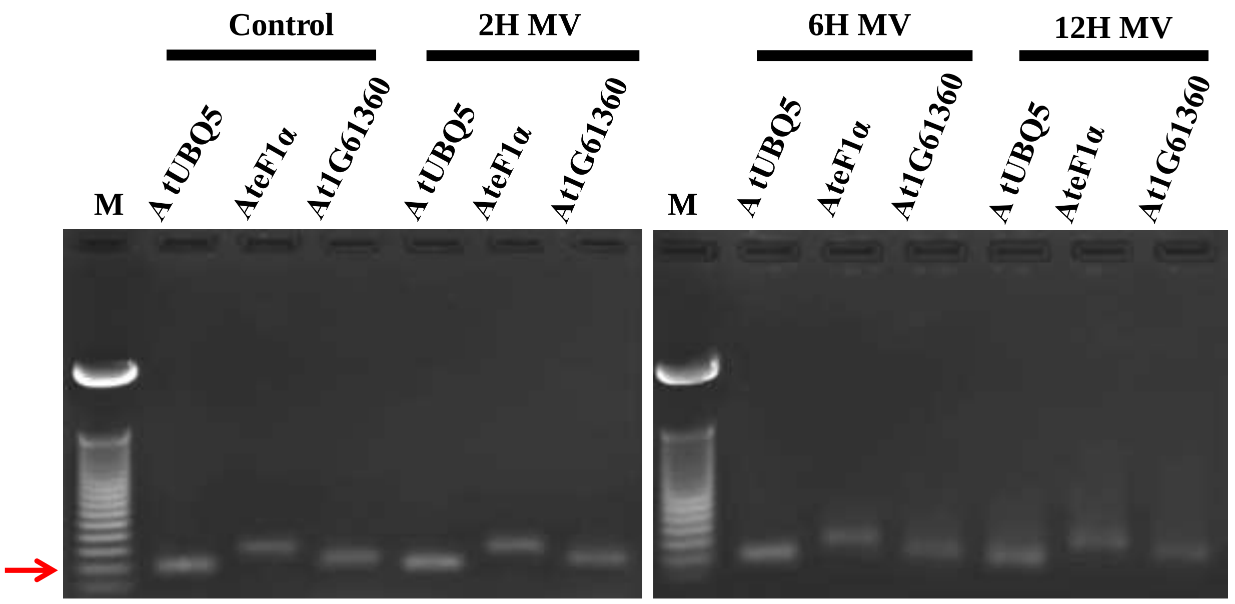

11. At1G61480

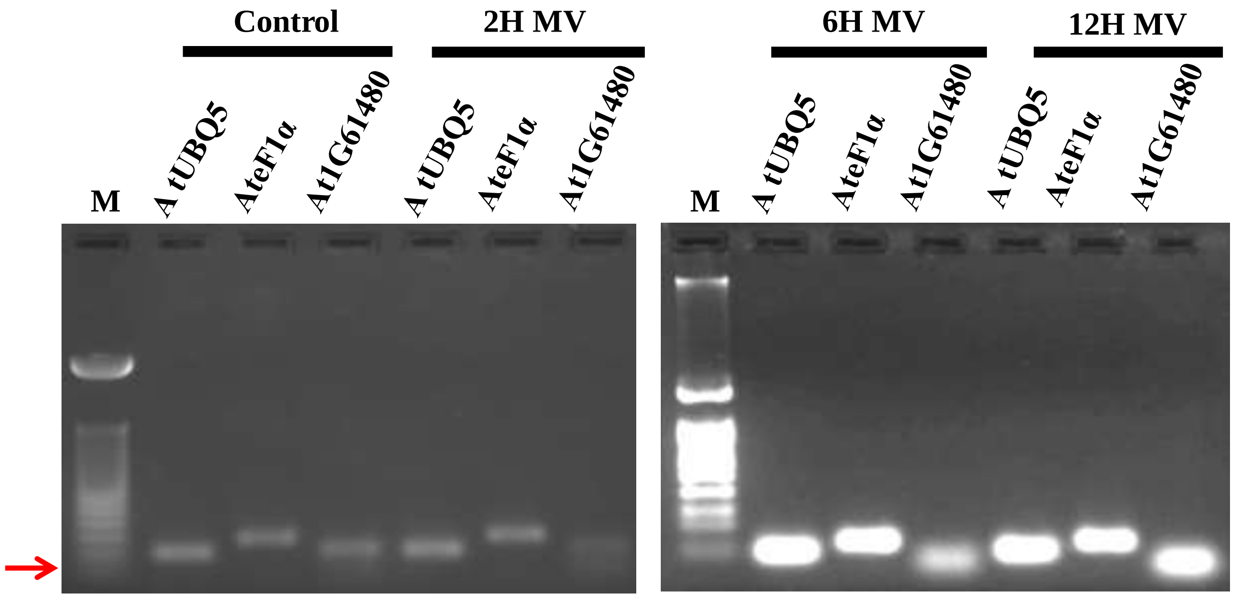

12. At1G61420

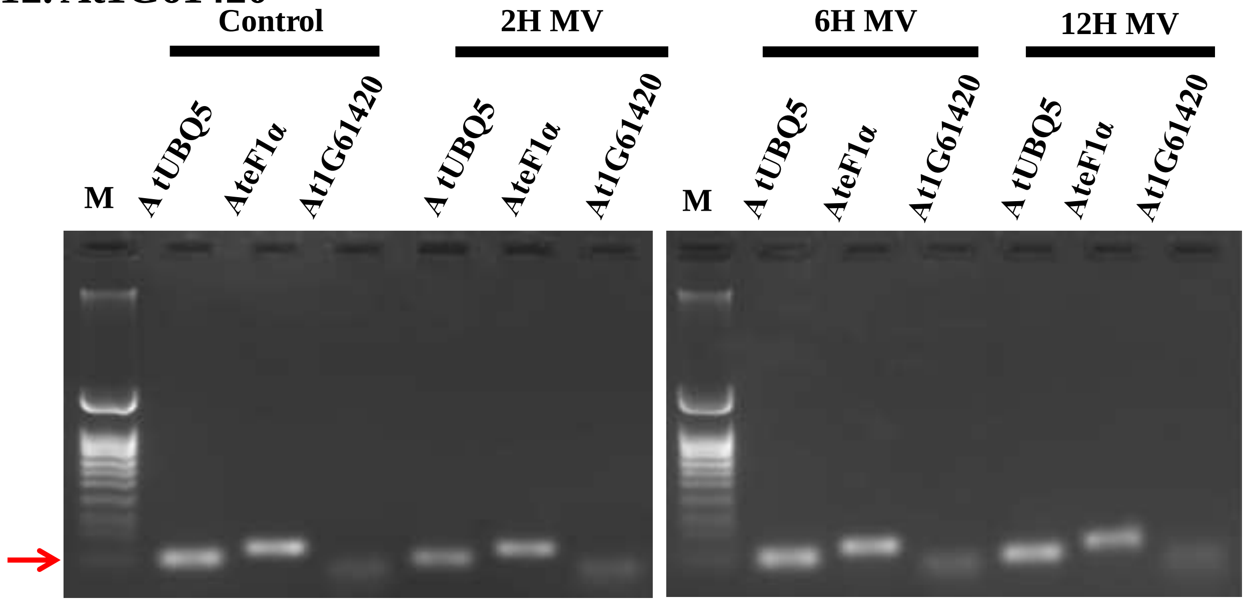

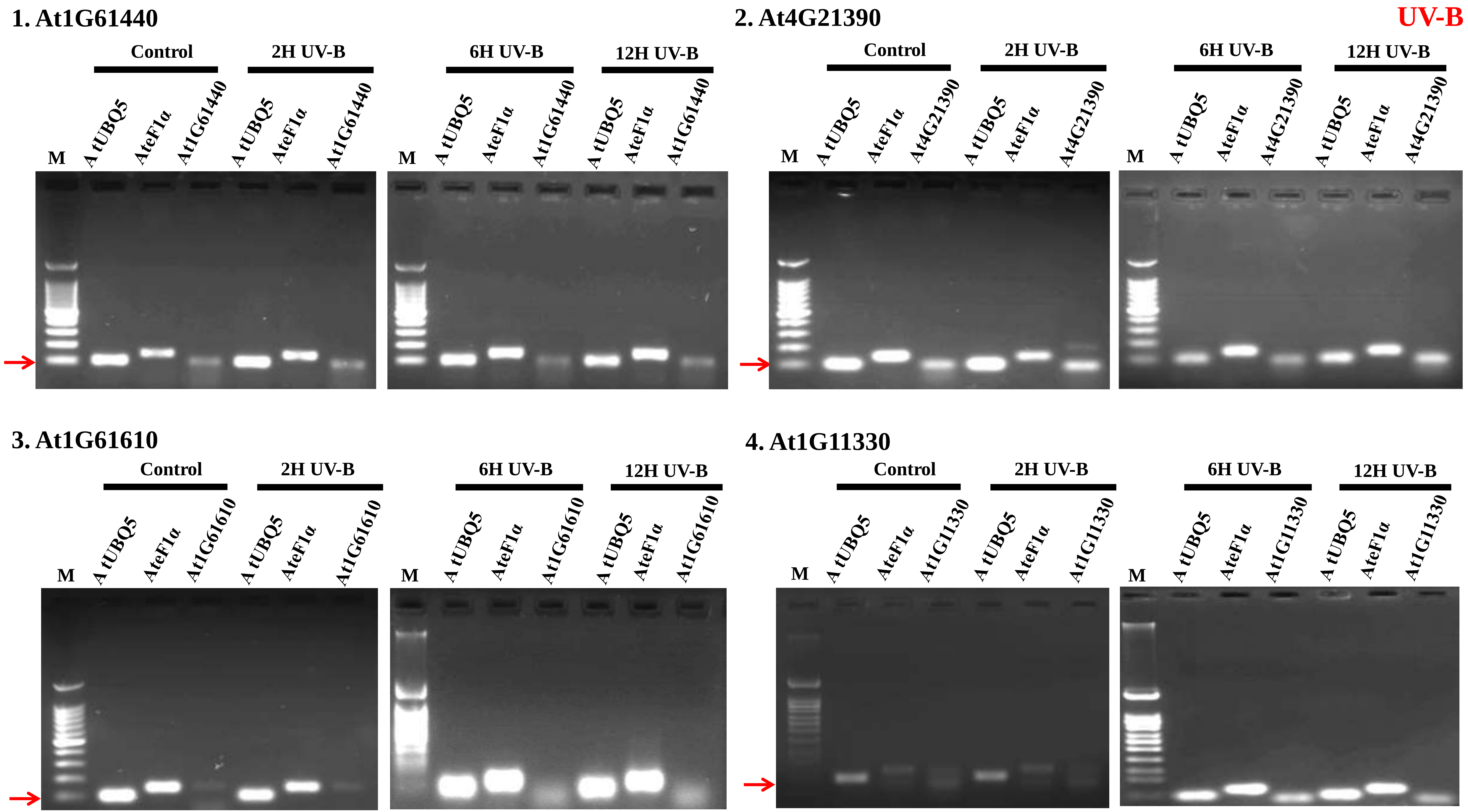

1. At1G61440
UV-B
Control
2H UV-B
6H UV-B
12H UV-B
M
A tUBQ5
AteF1α
At1G61440
2. At4G21390
Control
2H UV-B
6H UV-B
12H UV-B
M
A tUBQ5
AteF1α
At4G21390
3. At1G61610
Control
2H UV-B
6H UV-B
12H UV-B
M
A tUBQ5
AteF1α
At1G61610
4. At1G11330
Control
2H UV-B
6H UV-B
12H UV-B
M
A tUBQ5
AteF1α
At1G11330

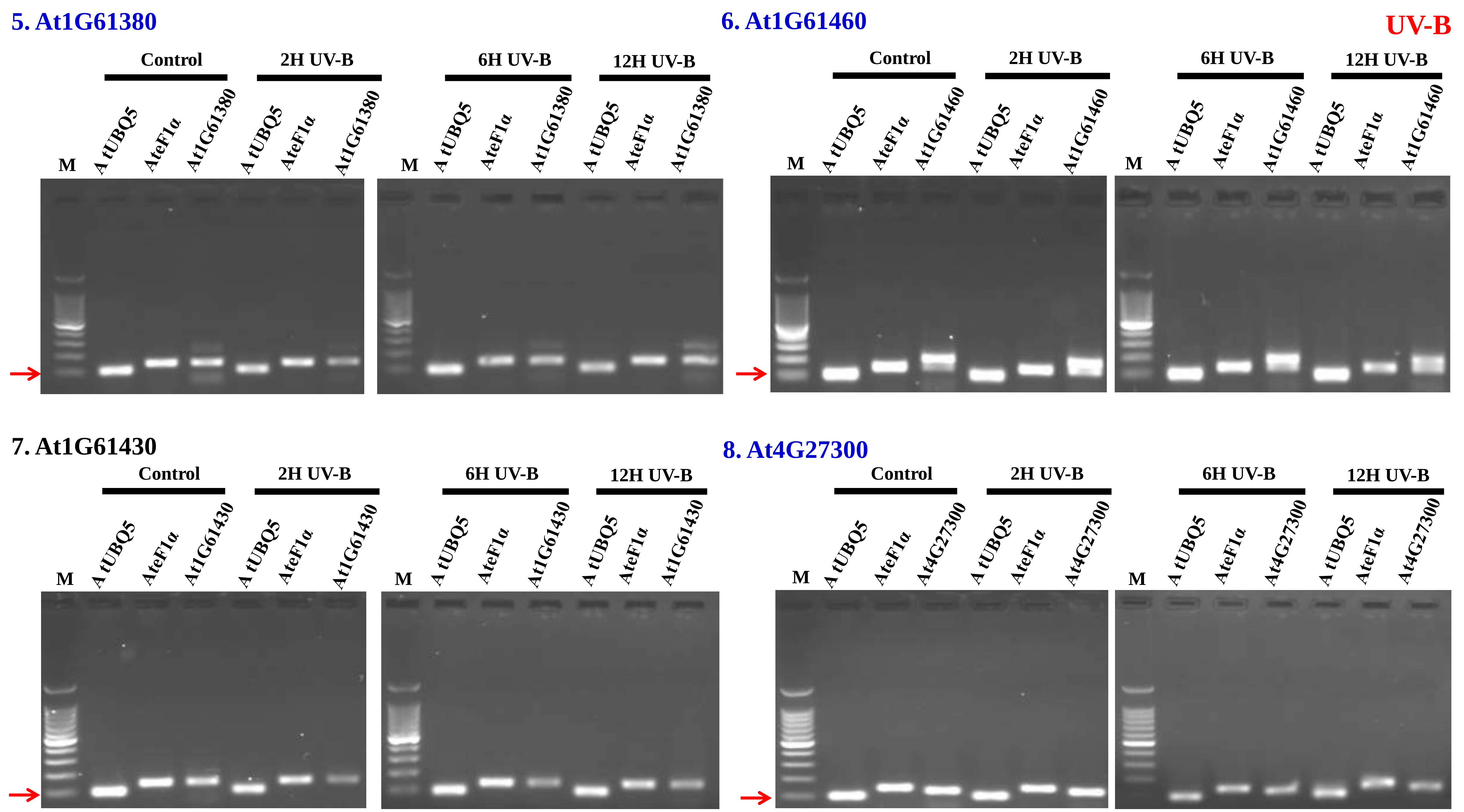

5. At1G61380
6. At1G61460
UV-B
Control
2H UV-B
6H UV-B
12H UV-B
M
A tUBQ5
AteF1α
At1G61380
At1G61460
7. At1G61430
8. At4G27300
At1G61430
At4G27300

9. At4G21380

10. At1G61360

UV-B

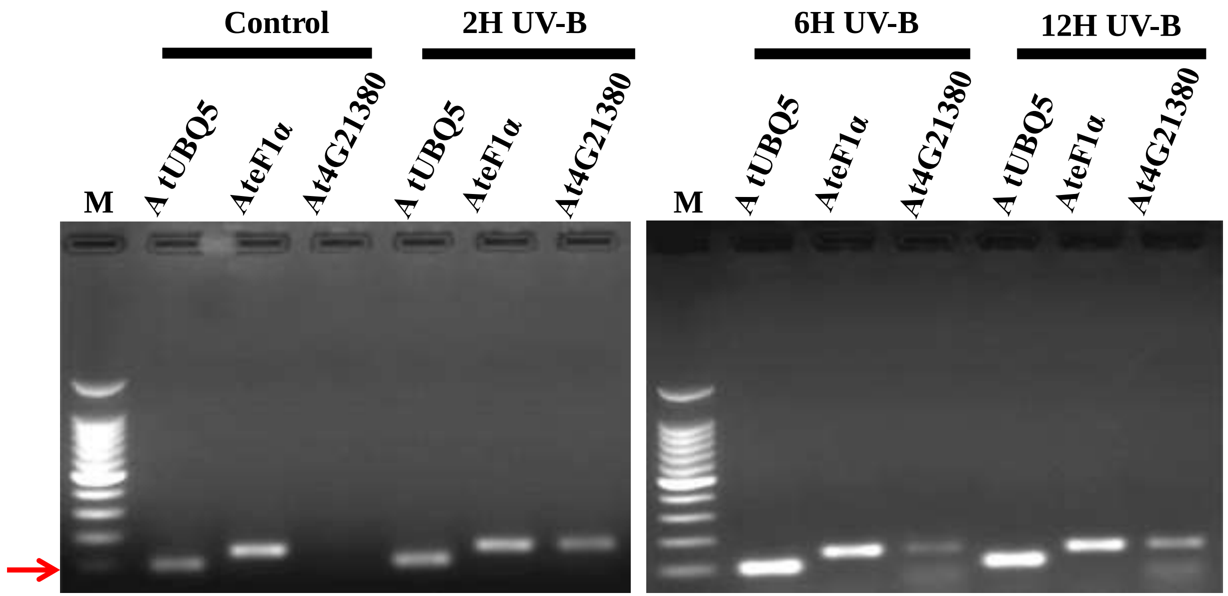

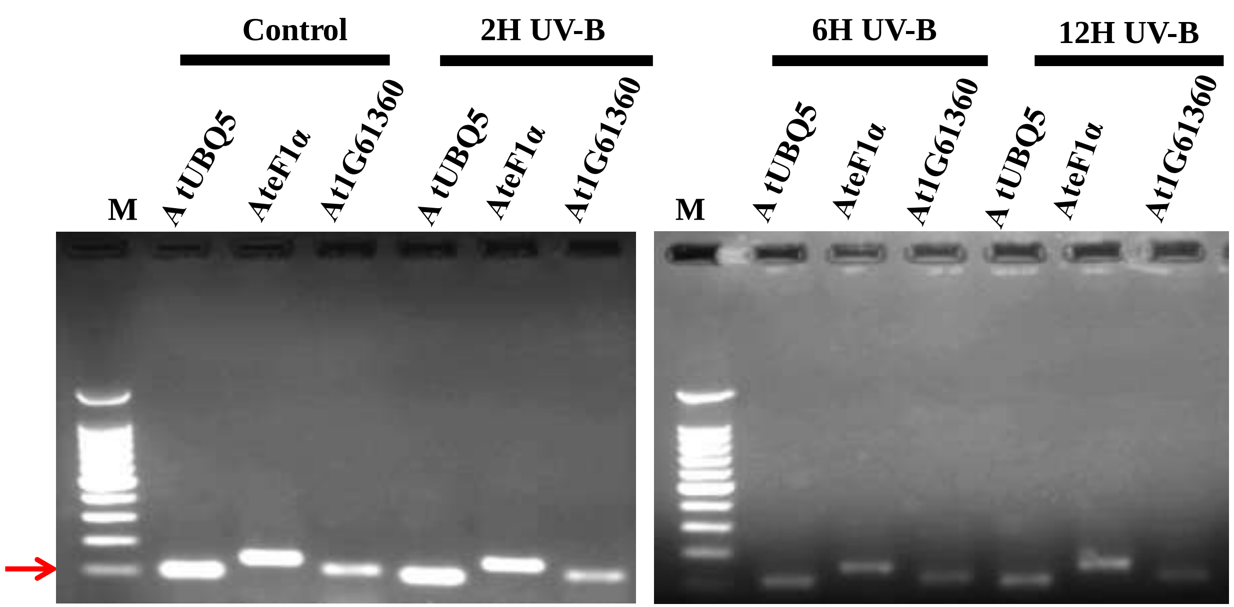

11. At1G61480

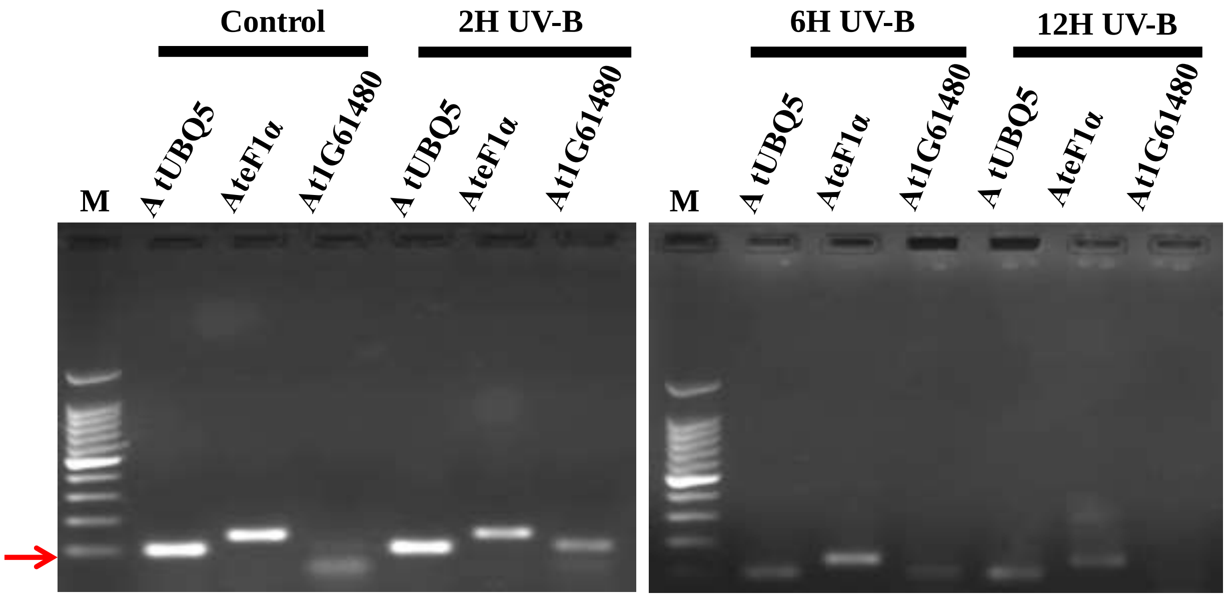

12. At1G61420

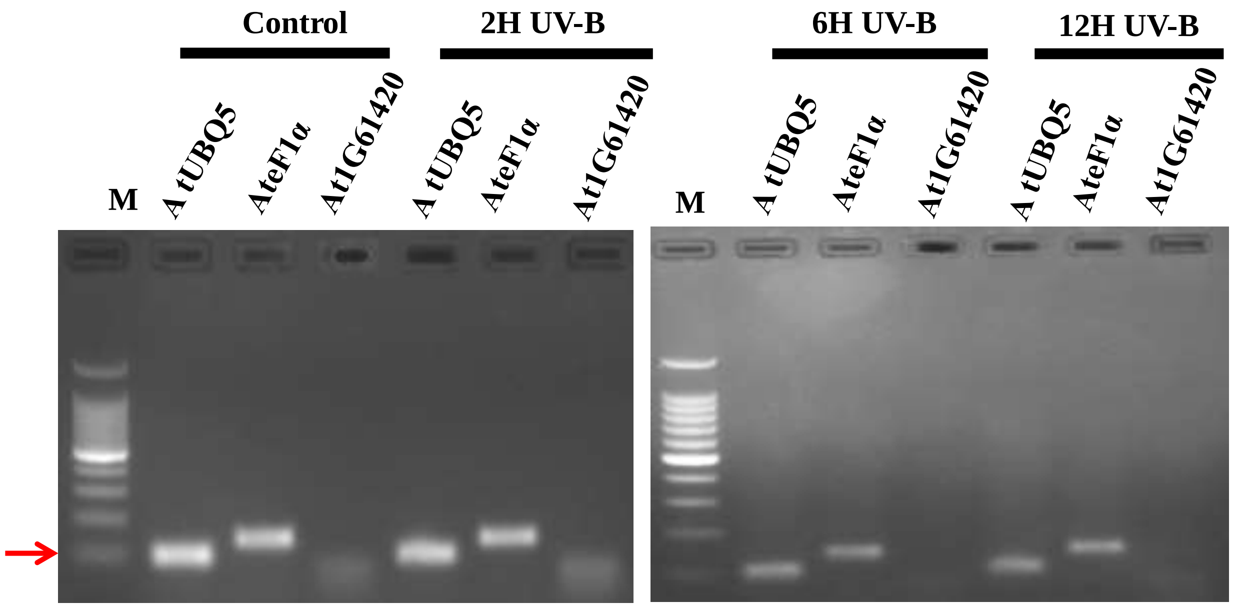

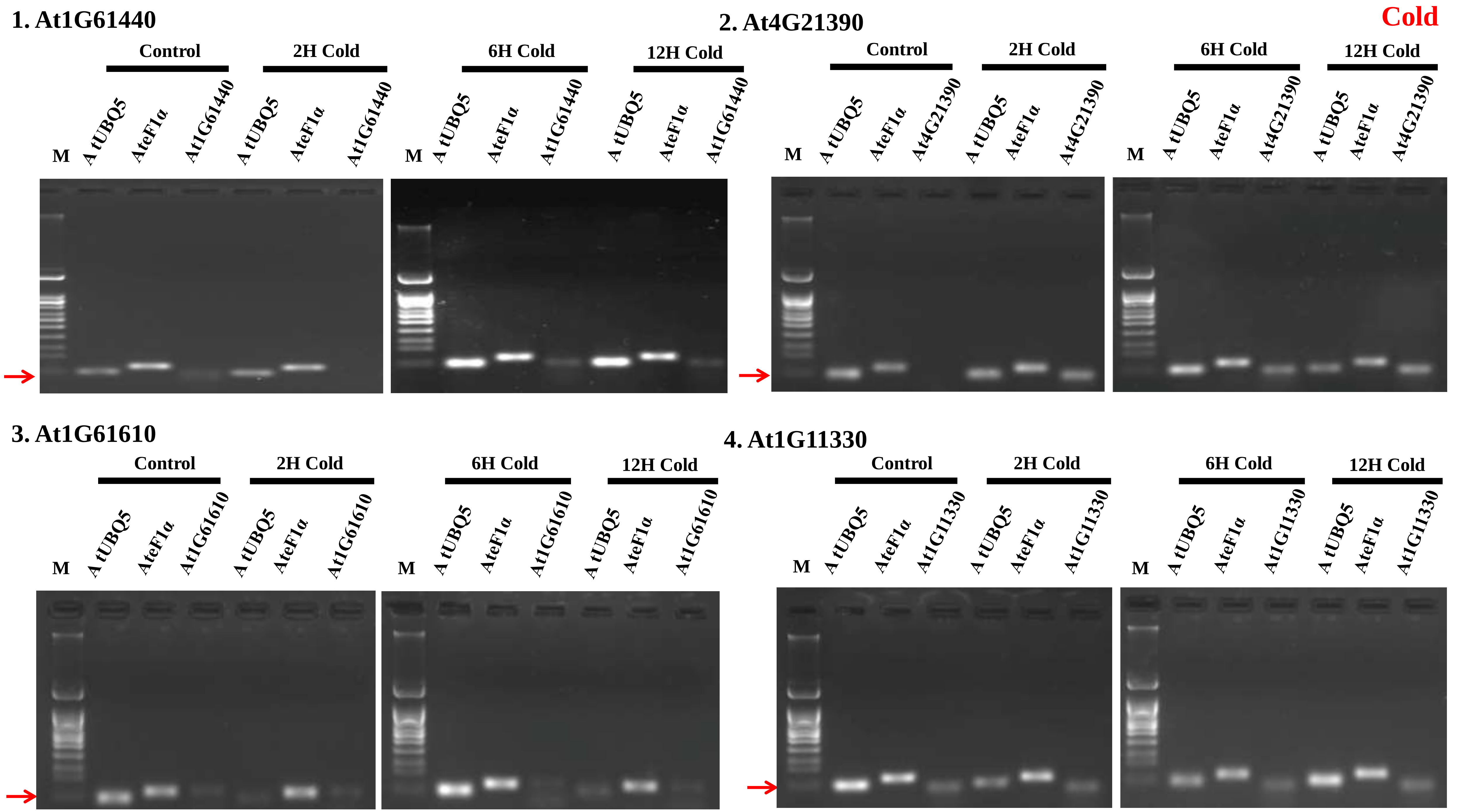

Cold
1. At1G61440
2. At4G21390
3. At1G61610
4. At1G11330
Control
2H Cold
6H Cold
12H Cold
M
A tUBQ5
AteF1α
At1G61440
At4G21390
At1G61610
At1G11330

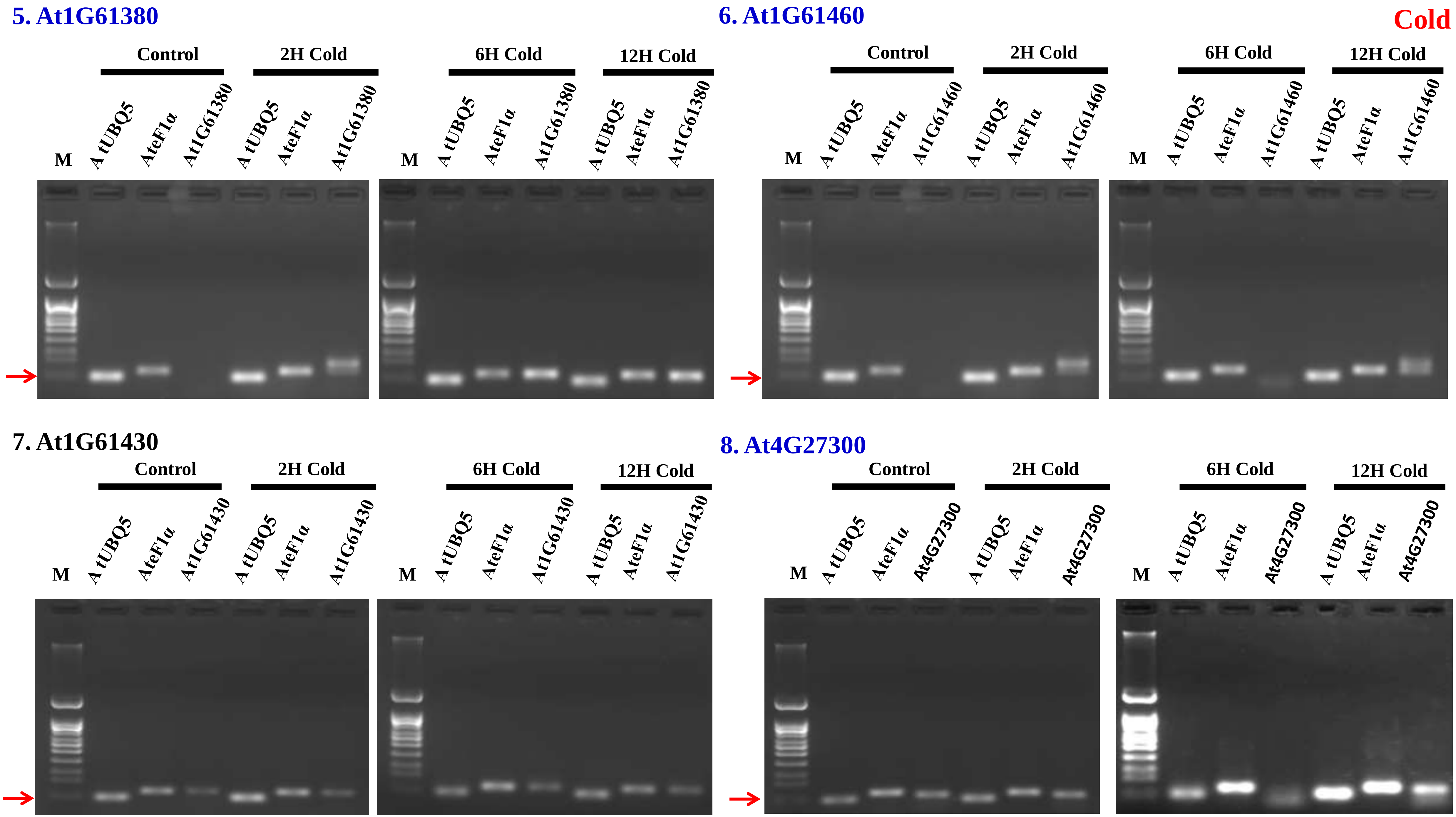
Cold
5. At1G61380
Control
2H Cold
6H Cold
12H Cold
M
A tUBQ5
AteF1α
At1G61380
6. At1G61460
Control
2H Cold
6H Cold
12H Cold
M
A tUBQ5
AteF1α
At1G61460
7. At1G61430
Control
2H Cold
6H Cold
12H Cold
M
A tUBQ5
AteF1α
At1G61430
8. At4G27300
Control
2H Cold
6H Cold
12H Cold
M
A tUBQ5
AteF1α
At4G27300

9. At4G21380

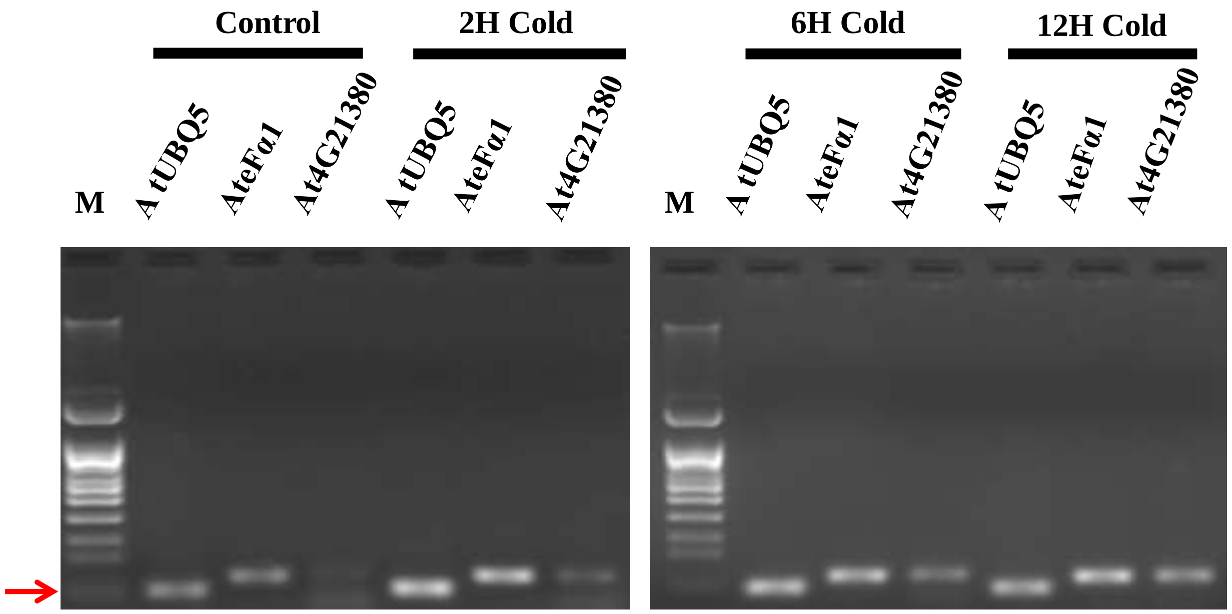

10. At1G61360

Cold

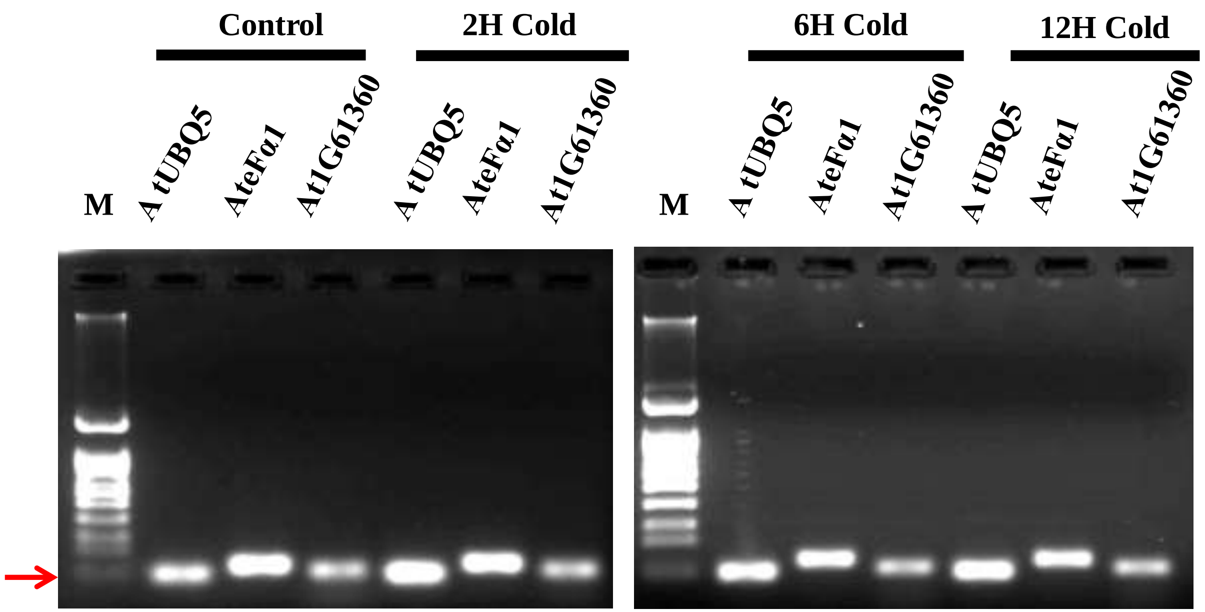

11. At1G61480

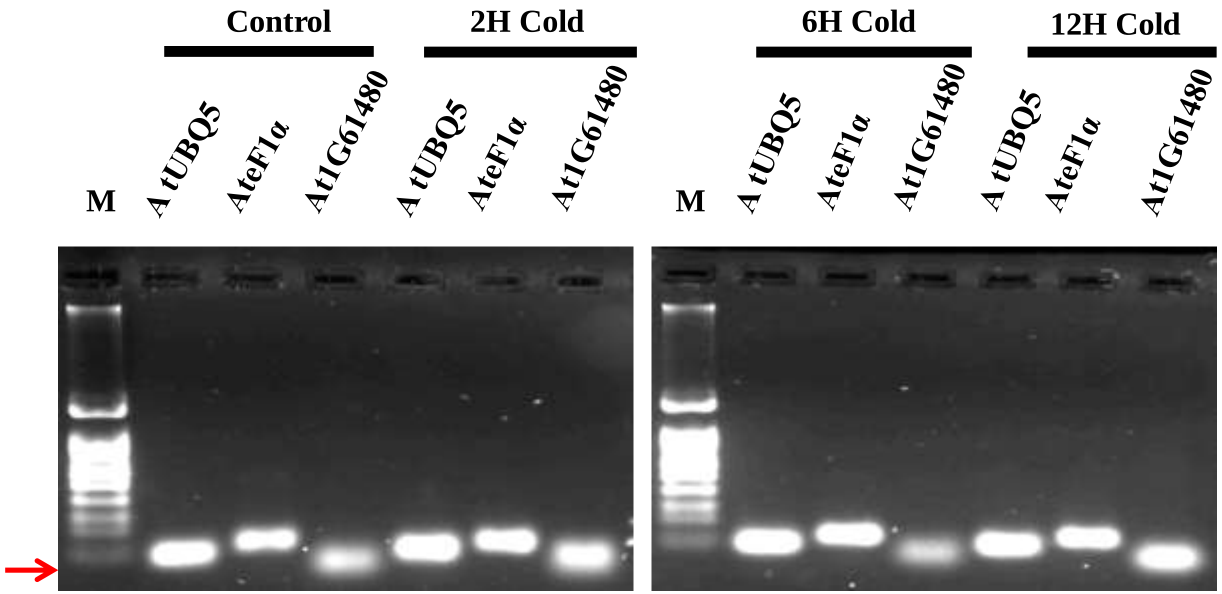

12. At1G61420

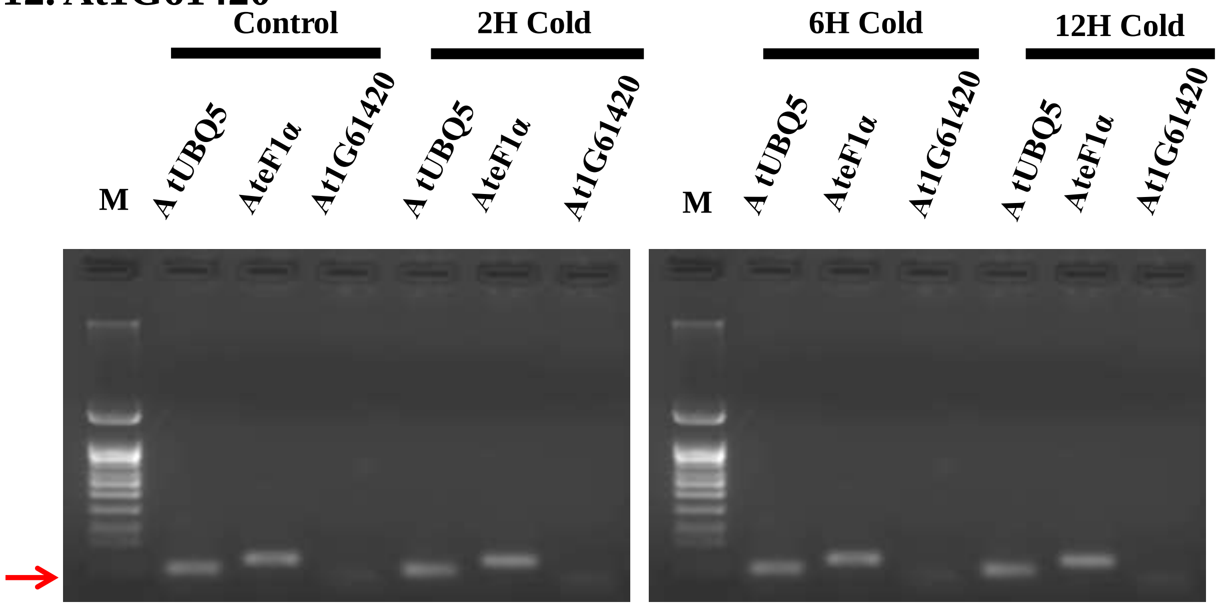

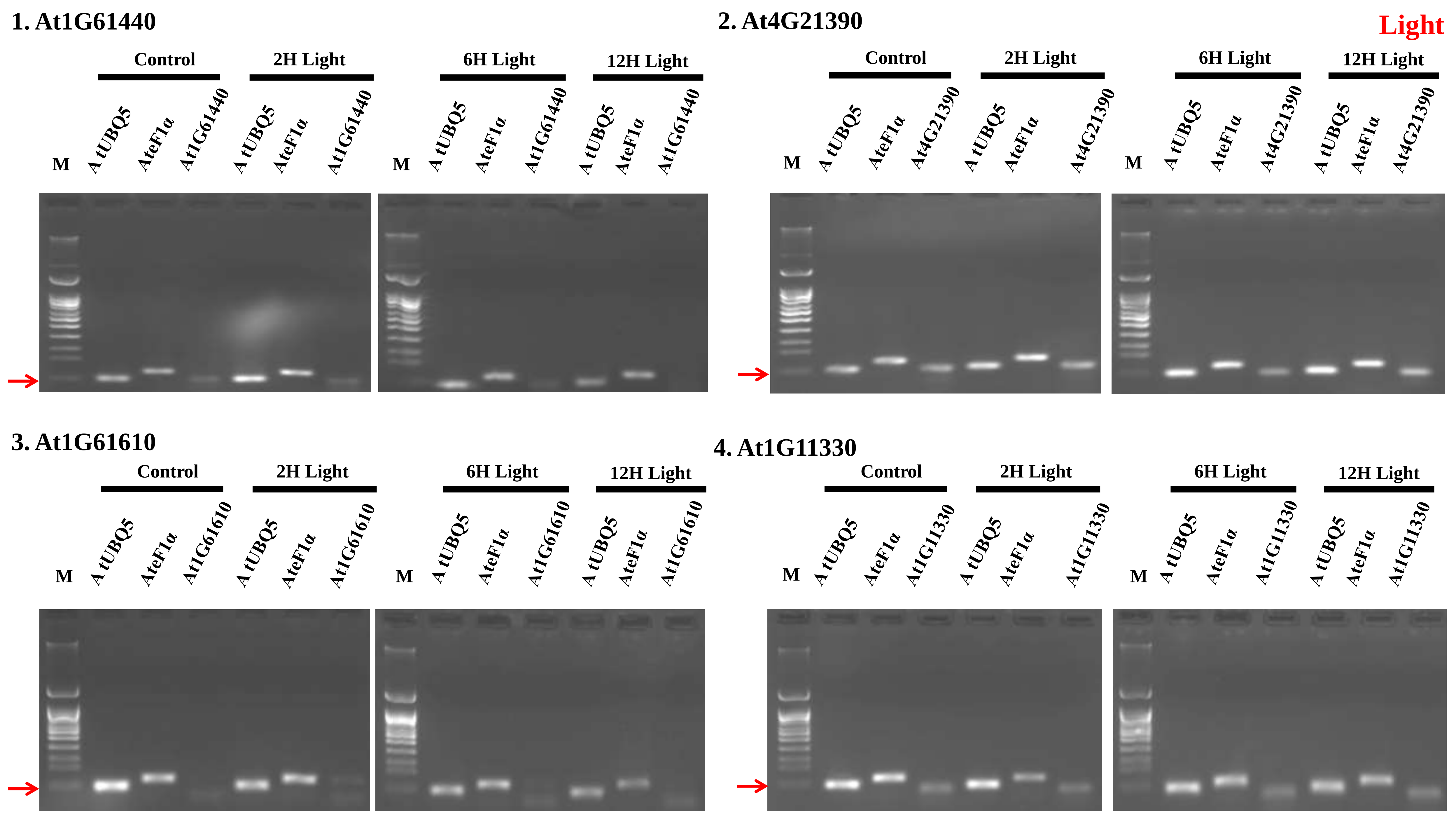

Light
1. At1G61440
Control
2H Light
6H Light
12H Light
M
A tUBQ5
AteF1α
At1G61440
2. At4G21390
Control
2H Light
6H Light
12H Light
M
A tUBQ5
AteF1α
At4G21390
3. At1G61610
Control
2H Light
6H Light
12H Light
M
A tUBQ5
AteF1α
At1G61610
4. At1G11330
Control
2H Light
6H Light
12H Light
M
A tUBQ5
AteF1α
At1G11330

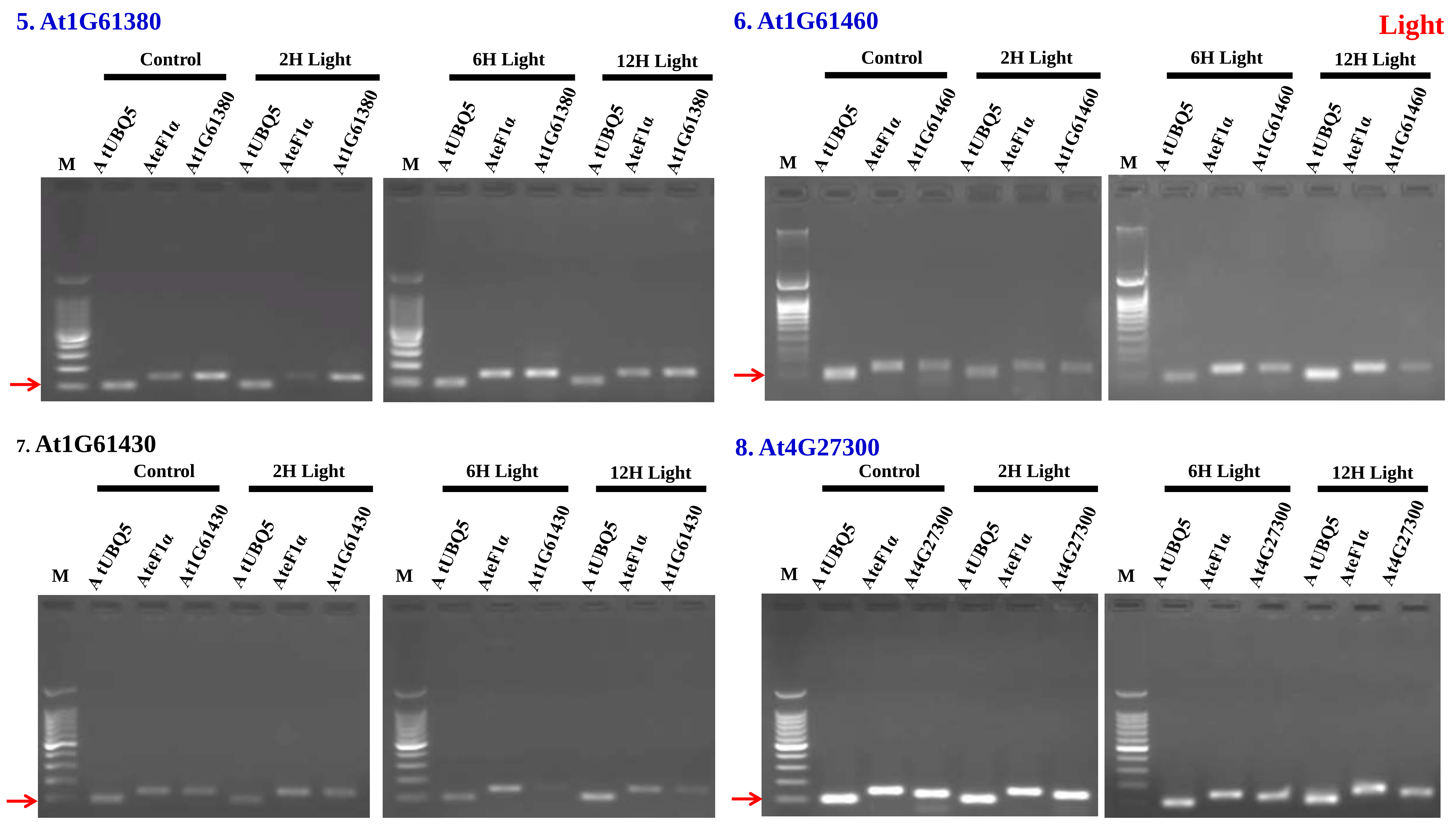

Light
5. At1G61380
Control
2H Light
6H Light
12H Light
M
A tUBQ5
AteF1α
At1G61380
6. At1G61460
Control
2H Light
6H Light
12H Light
M
A tUBQ5
AteF1α
At1G61460
7. At1G61430
Control
2H Light
6H Light
12H Light
M
A tUBQ5
AteF1α
At1G61430
8. At4G27300
Control
2H Light
6H Light
12H Light
M
A tUBQ5
AteF1α
At4G27300

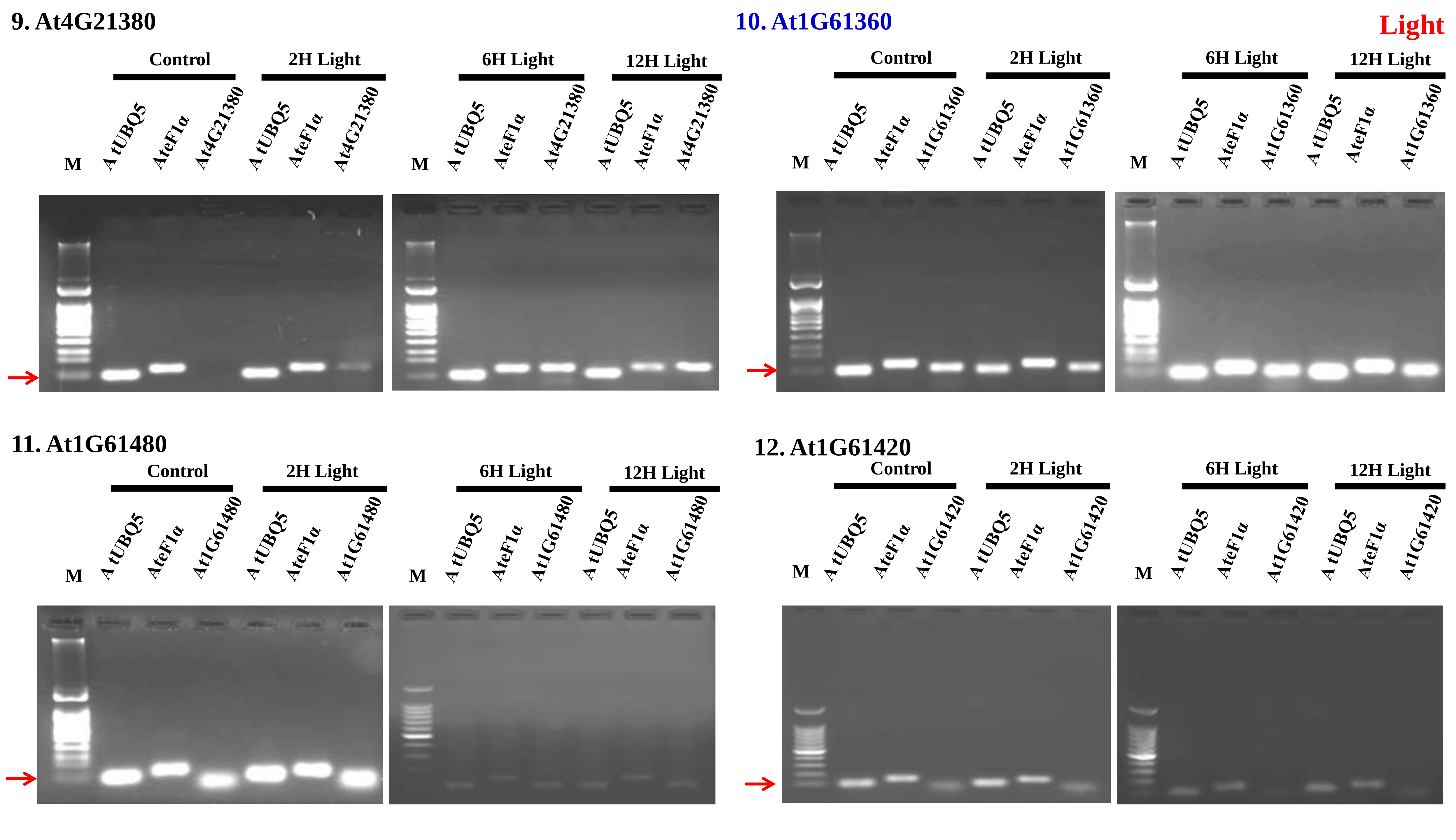

9. At4G21380
Control
2H Light
6H Light
12H Light
M
A tUBQ5
AteF1α
At4G21380
10. At1G61360
Light
Control
2H Light
6H Light
12H Light
M
A tUBQ5
AteF1α
At1G61360
11. At1G61480
Control
2H Light
6H Light
12H Light
M
A tUBQ5
AteF1α
At1G61480
12. At1G61420
Control
2H Light
6H Light
12H Light
M
A tUBQ5
AteF1α
At1G61420
